# An Overview of the Phytochemistry, Biological Activities and Therapeutic Potential of *Epimedium* spp.

**DOI:** 10.3390/plants15142114

**Published:** 2026-07-08

**Authors:** Ariana-Simina Friș, Irina Lazarova, Maya Georgieva, Loredana Gabriela Stana, Roxana Folescu, Ioana Zinuca Magyari-Pavel, Melania Munteanu, Corina Danciu

**Affiliations:** 1 Department of Pharmacognosy-Phytotherapy, “Victor Babes” University of Medicine and Pharmacy, Eftimie Murgu Square 2, 300041 Timisoara, Romania; ariana.fris@umft.ro (A.-S.F.); ioanaz.pavel@umft.ro (I.Z.M.-P.); corina.danciu@umft.ro (C.D.); 2 Research and Processing Centre of Medical and Aromatic Plants, “Victor Babes” University of Medicine and Pharmacy, Eftimie Murgu Square 2, 300041 Timișoara, Romania; 3 Doctoral School, “Victor Babes” University of Medicine and Pharmacy, Eftimie Murgu Square 2, 300041 Timișoara, Romania; 4 Department of Chemistry, Faculty of Pharmacy, Medical University of Sofia, 2 Dunav Str., 1000 Sofia, Bulgaria; lazarova@pharmfac.mu-sofia.bg; 5 Department of Pharmaceutical Chemistry, Faculty of Pharmacy, Medical University of Sofia, 2 Dunav Str., 1000 Sofia, Bulgaria; mgeorgieva@pharmfac.mu-sofia.bg; 6 Department I, Discipline of Anatomy and Embryology, “Victor Babes” University of Medicine and Pharmacy, 300041 Timisoara, Romania; 7 Department of Family Medicine, “Victor Babes” University of Medicine and Pharmacy, Eftimie Murgu Square 2, 300041 Timisoara, Romania; folescu.roxana@umft.ro; 8 Department of Pharmaceutical Sciences, Faculty of Pharmacy, “Vasile Goldiș” Western University of Arad, 310045 Arad, Romania; munteanu.melania@uvvg.ro

**Keywords:** *Epimedium*, horny goat weed, prenylated flavonoids, icariin, pharmacological activity, phytochemistry, antioxidant, anticancer, pharmacokinetics, toxicology

## Abstract

The genus *Epimedium* L. comprises a group of perennial herbs widely distributed across East Asia, with five representative species that include *Epimedium grandiflorum* C. Morren, *Epimedium brevicornu* Maxim., *Epimedium sagittatum* (Sieb. et Zucc.) Maxim., *Epimedium koreanum* Nakai. and *Epimedium pubescens* Maxim. For centuries, these species have been used in traditional Chinese medicine for their aphrodisiac, anti-osteoporotic and estrogen-like properties in the treatment of erectile dysfunction, osteoporosis, rheumatoid arthritis and menopausal symptoms. The aim of this review is to present an extensive and updated synthesis of the phytochemistry, biological activities and therapeutic properties of these five species by examining the relationship between the phytochemical composition and their pharmacological properties. Phytochemical analyses indicate that *Epimedium* spp. are defined by their significant content of prenylated flavonol glycosides, including icariin, icaritin, icariside, baohuoside and epimedin A-C. Recent studies have confirmed that these compounds are responsible for the genus’ therapeutic potential. They possess a variety of effects, ranging from aphrodisiac and antioxidant properties to anti-inflammatory and immunomodulatory activities, as well as neuroprotective, cardioprotective and anticancer benefits. Although preclinical findings are increasingly compelling, robust clinical evidence is still lacking for all five species. Taken together, the data summarized here position *Epimedium* spp. as a valuable source of bioactive prenylated flavonoids, while underscoring that standardized methodologies and rigorous clinical trials are essential to translate this potential into validated therapeutic applications.

## 1. Introduction

Phytotherapy, once rooted exclusively in traditional healing remedies, has undergone a profound scientific metamorphosis over the past few years [1]. Plant-based medicine, once regarded primarily as empirical or folkloric, has become a vital resource for developing new drugs thanks to advances in phytochemistry, pharmacology and molecular biology [2]. The renewed interest in phytotherapy largely stems from the recognition of medicinal plants as a valuable source of bioactive compounds with complex synergistic actions that frequently exceed those of synthetic drugs [3]. In retrospect, a major limitation of traditional herbal remedies has been the lack of standardization and the uncertainty surrounding their pharmacokinetic properties. In recent years, advances in analytical standardization have enabled more precise phytochemical characterization, while modern drug-delivery technologies have provided new strategies to overcome pharmacokinetic limitations [4]. In particular, advances in formulation, such as nanoparticles, nanocrystals, nanoemulsions, phytosomes and liposomal delivery systems, have helped overcome these limitations by increasing the bioavailability of phytoconstituents in order to ensure predictable therapeutic effects [5].

Medicinal plants have long been recognized as an invaluable source of pharmacologically active molecules with numerous modern drugs being derived from phytoconstituents [6]. Among the most notable examples are: paclitaxel, a tetracyclic diterpenoid with antineoplastic properties extracted from the bark of *Taxus brevifolia* Nutt. [7]; vincristine and vinblastine, alkaloids used as anticancer agents isolated from *Catharanthus roseus* (L.) G. Don; galantamine, an alkaloid obtained from *Galanthus nivalis* L., an acetylcholinesterase inhibitor used to delay cognitive decline in Alzheimer’s disease [8]; digoxin, a cardiac glycoside isolated from *Digitalis lanata* Ehrh. used in heart failure and fibrillation [9]; morphine, an opiate isolated from *Papaver somniferum* L. [10] as a potent analgesic [11]; artemisinin, isolated from *Artemisia annua* L. [12]; and quinine extracted from *Cinchona officinalis* L. [13] used as antimalarial agents.

The genus *Epimedium* L. has attracted considerable scientific interest through its biological activities, assigned mainly to prenylated flavonoids [14]. *Epimedium* folium, known as “Xiang Ling pi” or “nine leaves on three stems” is an herbal remedy obtained from the dried leaves of 4 *Epimedium* species, *Epimedium brevicornu* Maxim., *Epimedium sagittatum* (Sieb. et Zucc.) Maxim., *Epimedium koreanum* Nakai. and *Epimedium pubescens* Maxim. The mix was first documented in Shennong Materia Medica Classic. *Epimedium* folium is used in China, Japan and South Korea for its medicinal benefits such as improvement of erectile function, strengthening bone and muscle, rheumatic pain alleviation. For centuries, these species have been utilized for the treatment of sexual dysfunction, kidney-yang deficiency, rheumatoid arthritis and osteoporosis [15]. The 2020 edition of the Chinese Pharmacopeia officially recognizes five *Epimedium* species: *Epimedium brevicornu* Maxim., *Epimedium sagittatum* (Sieb. et Zucc.) Maxim., *Epimedium koreanum* Nakai., *Epimedium pubescens* Maxim. and *Epimedium wushanense* T. S. Ying [16]. All five species are commonly known as bishop’s hat, fairy wings, Rowdy lamb herb, barrenwort, yin yang huo or horny goat weed. In addition to the *Epimedium* species mentioned above, this review also covers *Epimedium grandiflorum* C. Morren, a species studied for its pharmacological properties and therapeutic potential [17].

More than 130 secondary metabolites have been identified in *Epimedium* folium, which include prenylated flavonoids, polysaccharides and organic acids, among which prenylated flavonoids exhibit notable pharmacological activity [15]. Icariin and its principal metabolite, icaritin, are the most studied bioactive compounds of *Epimedium* spp. These phytoconstituents have shown anti-inflammatory and immunomodulatory activities, by modulating pro-inflammatory factors such as TNF-α, IL-6, and NF-κB signaling pathway, with demonstrated therapeutic potential in neurodegenerative diseases, cardiovascular diseases and several types of cancer [18,19].

Despite the growing body of evidence supporting the pharmacological proprieties of *Epimedium* spp. extracts and their prenylated flavonoids content, the existing literature is still fragmented, and extensive evaluations that integrate its phytochemical diversity with mechanistic insights are limited.

The aim of the systematic review is to provide a comprehensive analysis of the recent evidence on the phytochemical composition, biological activities and therapeutic potential of five *Epimedium* species: *Epimedium grandiflorum*, *Epimedium brevicornu*, *Epimedium sagittatum*, *Epimedium koreanum*, *Epimedium pubescens*. This systematic review consolidates recent advances in analytical chemistry, molecular pharmacology and experimental therapeutics to identify the principal bioactive compounds, focusing on prenylated flavonoids, and to identify their mechanisms of action, thereby providing a framework for future preclinical and clinical investigations.

## 2. Phytochemistry of *Epimedium* spp.

More than 270 compounds have been isolated from the genus *Epimedium*, among which prenylated flavonoids represent the principal bioactive metabolites that largely account for their therapeutic potential for bone health, sexual function, and aging processes [20]. Other classes of compounds reported in the selected *Epimedium* species include ionones, lignans and their glycosides, sesquiterpenes and their glycosides, phenolic glycosides, phenylethanoids and their glycosides, and others. The phytochemical composition of *Epimedium* varies across its different parts, with different species exhibiting distinct compound profiles [21]. The prenylflavonoid glycosides and their main representatives, icariin, epimedin A, B and C, are the main bioactive principles found in *Epimedium* spp. and are known as the principal biologically active components and chemotaxonomic markers in Epimedium crude plant extracts [22]. Detailed phytochemical analyses of the Epimedium extracts have been performed using various chromatographic techniques with Sephadex LH-20, silica gel, RP-18 silica and preparative TLC, resulting in the isolation of pure compounds. Their structures were elucidated using UV, IR, 1D and 2D NMR spectroscopic techniques, as well as the HRESIMS methods. Chemical transformations have also been employed (Table 1).

## 3. *Epimedium grandiflorum* C. Morren

### 3.1. Phytochemistry

#### 3.1.1. Prenylated Flavonoids

**Figure 1:**
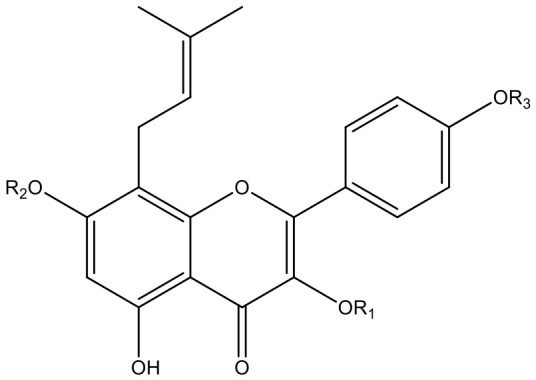
General chemical structure of prenylflavonol derivatives.

**Figure 2:**
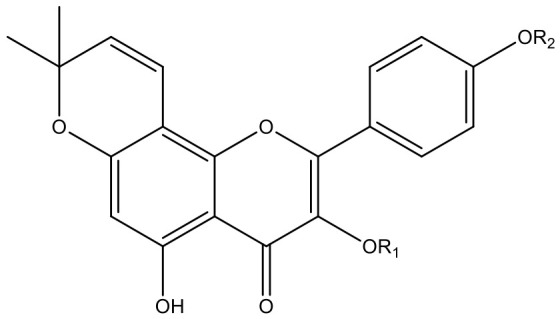
General structure of pyranoflavonol derivatives.

Prenylated flavonoids constitute one of the major classes of bioactive compounds that exhibit a flavonoid skeleton (2-phenylchromen-4-one) and a lipophilic prenyl group. They exhibit a wide range of biological properties, including cytotoxic, anti-inflammatory, neuroprotective, antimicrobial, antioxidant, anti-diabetic, estrogenic, cardioprotective and enzyme inhibitory effects [23]. These substances are more lipophilic than the parent flavonoids without prenylation, which gives them a higher affinity for P-glycoprotein in cell membranes and makes them potential P-glycoprotein inhibitors [24]. In particular, 32 flavonoids isolated from *Epimedium grandiflorum* C. Morren, belong to the 8-prenylated flavonol subclass (Figure 1 and Figure 2). Structurally, they are characterized by the presence of a prenyl substituent in the 8 position, a free –OH group or a –OH group incorporated with sugar in positions C-3 and C-7, a free –OH group in the 5 position, and a methoxy or hydroxyl moiety in the 4′-C position of the flavonoid rings [25]. According to the literature, the C-8 prenyl group is a key feature of both cytotoxic and osteogenic activity, contributes to anti-inflammatory and enzyme inhibitory effects. Cyclisation of the C-8 prenyl substituent significantly reduces cytotoxicity and affects the inhibition of TNF-α, COX and NO production. The presence of a free -OH group at position C-3 contributes to cytotoxic activity, but glycosylation could lower the activity. The presence of a hydroxyl group at position C-4′ enhances both cytotoxic and anti-inflammatory activity. Furthermore, the presence of –OH groups at positions C-5 and C-7, as well as a keto group at position C-4, enhances the anti-inflammatory effects. Characteristics favorable to better vasorelaxant activity in prenylated flavonoids include prenylation of the A-ring and –OH groups at positions C-2′ and C-4′ of the B-ring. Structural features associated with tyrosinase inhibitory activity include the presence of hydroxyl groups at positions C-5, C-7, C-2′ and C-4′ [26]. Regarding neuraminidase inhibition, the presence of hydroxyl groups at C-3′ and C-4′ are beneficial. Isolated from *Epimedium grandiflorum* constituents epimedigrandioside A, korepimeoside B, korepimeoside A, epimedoside and icariside II have demonstrated cytotoxic activity against cancer cell lines—SK-MEL, KB, BT-549, SK-OV-3 and noncancerous kidney cell lines LLC-PK1 and VERO with the IC_50_ values between 9 and 45 μM. Most of the 8-Prenylated flavonols present in *Epimedium grandiflorum* methanolic extract possess anti-inflammatory activity. The most prominent of them are epimedigrandioside A, korepimeoside B, korepimeoside A, icariside II, and desmethylicaritin because they inhibit both NF-κB and iNOS, with the IC_50_ values in the range of 14–26 μM [27].

#### 3.1.2. Megastigmanes (Ionones)

**Figure 3:**
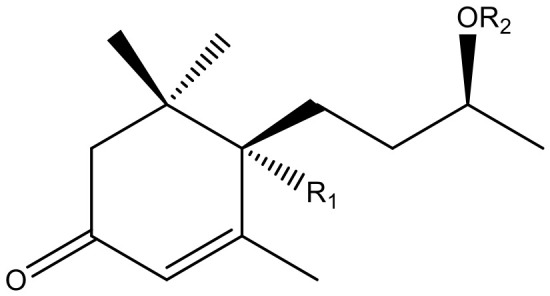
General structure of megastigman-4-en-3-one derivatives.

**Figure 4:**
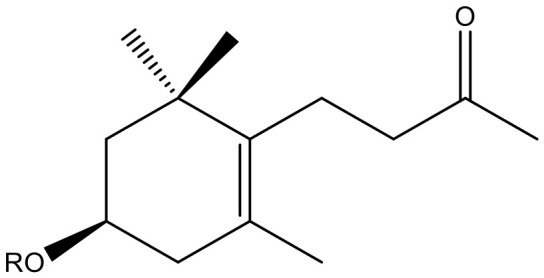
General structure of 3-hydroxy-dihydro-β-ionone derivatives.

**Figure 5:**
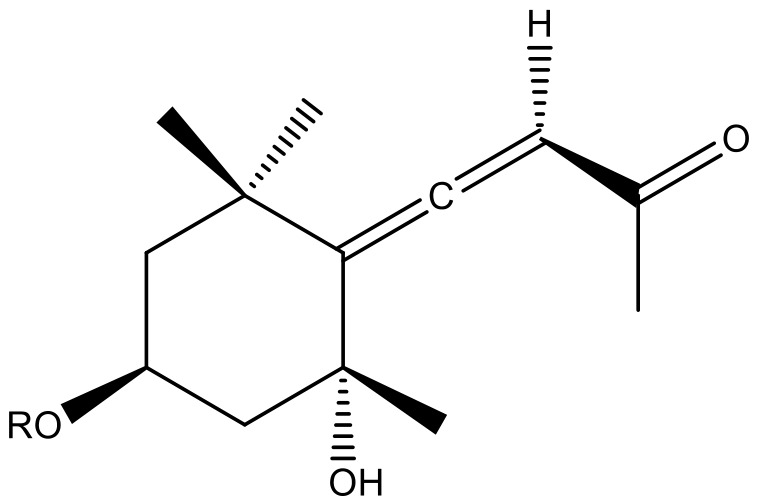
General structure of allene-possessing megastigmane derivatives.

**Figure 6:**
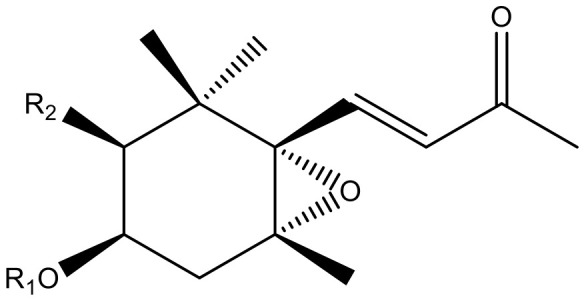
General structure of epoxy-ionone megastigmanes.

Seventeen ionones have been isolated from the *Epimedium grandiflorum* plant. These compounds belong to a large group called C_13_-norisoprenoid megastigmanes. The megastigmanes are subdivided into 17 classes. These secondary compounds originate from the oxidative cleavage of carotenoids, and have potential applications in the food and pharmaceutical industries as flavoring agents [28]. The biological properties described in the literature include antioxidant, anti-inflammatory, anti-elastase, cytotoxic and tyrosinase inhibitory activities [29]. The megastigmanes identified in *Epimedium grandiflorum* are classified as follows: (6S,9R)-Roseoside II, 9-epi-Blumenol B, icariside B_5_, 9-epi-Blumenol C, Blumenol C 9-O-β-D-glucopyranoside are in the α-ionol megastigmane class (Figure 3); (-)-3-hydroxy-dihydro-β-ionone, icariside B_6_, icariside B_7_, icariside B_4_ are in the β-ionone megastigmane class (Figure 4); grasshopper ketone and icariside B_1_ are in the allene-possessing megastigmane class (Figure 5); and icariside B_3_ aglycone, icariside B_3_, icariside B_2_ aglycone, icariside B_2_ and 3β-Acetoxy-5α,6α-epoxy-β-ionone are in the epoxy-β-ionone megastigmane class (Figure 6), while icariside B_10_ belongs to the α-ionone megastigmane class [30]. The compound (6S,9R)-Roseoside II reveals strong anti-inflammatory activity by inhibiting the LPS-induced NO release by macrophages with an IC_50_ value of 7.31 μM [31]. The SAR analysis defined that the saturation at the C-7 and C-8 positions in the side-chain, the presence of a double bond conjugated with a C=O group and the sugar substituent at the C-9 position play a crucial role in the discussed prominent effect. Both icariside B_5_ and blumenol C 9-O-β-D-glucopyranoside also meet the structural criteria, except for the double bond at C-7, and exhibit anti-inflammatory activity. Regarding the anti-allergic properties of Roseoside II, the presence of an enone functional group and double bond at C-7 position in the side-chain determined considerable inhibition effect on histamine release from RBL-2H3 cells and rat peritoneal exudate cells [32]. Icariside B_2_ is one of the main representatives of epoxy-β-ionone megastigmanes, which are isolated in *Epimedium grandiflorum* for the first time, and after that they were demonstrated in the other species. Icariside B_2_ has anti-inflammatory effects by inhibiting MAPK/NF-κB pathways, reducing pro-inflammatory cytokines (e.g., TNF-α, IL-6, IL-1β), and decreasing iNOS and COX-2 expression. The compound is a mild inhibitor on both adipocyte differentiation and pancreatic lipase. Its anti-edema activity in carrageenan-induced mice has also been studied [33]. The presence of the 3,6-epoxy ring system appears to play a key structural role for the anti-inflammatory effect of the compound.

#### 3.1.3. Other Compounds

Lignans are a class of secondary metabolites, consisting of two phenyl-propanoid molecules connected by 8–8′ carbon atoms. To date, twelve lignans and their glycosides have been identified in *Epimedium grandiflorum*.

Sesquiterpene glycosides are a class of secondary metabolites formed by attaching one or more sugar molecules to a 15-carbon terpene backbone consisting of three isoprenoid units. Four sesquiterpene glycosides have been isolated from *Epimedium grandiflorum* species: ikarisoside C_1_, ikarisoside C_2_, ikarisoside C_3_, and ikarisoside C_4_.

The presence of six phenol glycosides (icariside A_1_, icariside A_2_, icariside A_3_, icariside A_4_, icariside A_5_ and icariside A_6_) has been proven in plant extracts from aerial parts of *Epimedium grandiflorum*. Five derivatives of phenethyl alcohol (tyrosol, phenethyl glucoside, icariside D1, salidroside and thalictoside) have been identified in *Epimedium grandiflorum* plant material.

Seven additional compounds have been identified in *Epimedium grandiflorum*, along with the previously mentioned constituents.

### 3.2. Biological Activities and Therapeutic Potential

#### 3.2.1. Antioxidant Activity

Asif et al. investigated the antioxidant potential of *Epimedium grandiflorum* through physicochemical characterization and in vitro evaluation [34]. The tested extracts were obtained from powdered leaves and solvents such as methanol, chloroform, ethyl acetate and n-hexane via hot extraction, as well as an aqueous extract prepared via cold extraction. The *Epimedium grandiflorum* leaves were purchased from China. The methanolic extract demonstrated substantial radical scavenging activity, as evidenced by standard antioxidant techniques such as DPPH, phosphomolybdenum and ferric reducing assays, suggesting a strong electron-donating capacity. The concentrations of the tested extracts were not clearly reported, which prevents a quantitative comparison with the other studies [34]. In an in vivo study, Munir et al. evaluated the antioxidant potential of a crude hydroethanolic leaf extract of *Epimedium grandiflorum* in male albino rats subjected to CCL4-induced oxidative stress [35]. The leaves were collected from a local market from Faisalabad, Pakistan. The extract was administered orally at doses of 50, 100 and 200 mg/kg body weight for 42 days. The treatment resulted in a dose-dependent antioxidant capacity, as evidenced by increased activities of superoxide dismutase (SOD) and catalase (CAT), and reduced malondialdehyde (MDA) levels [35]. Ilkhani et al. conducted an in vivo study investigating the antioxidant effects of a crude hydroethanolic extract of *Epimedium grandiflorum*, the leaves for the extract were purchased from a local market in Teheran, Iran [36]. Using a rat model of experimentally induced cryptorchidism, the extract was administered orally at doses of 100, 200 and 400 mg/kg for up to 28 days. Following the treatment, MDA levels were significantly reduced, while key antioxidant enzymes like SOD and glutathione peroxidase (GPx), were increased. These findings indicate that oxidative stress in testicular tissue was attenuated. The highest dose (400 mg/kg) produced the most significant biochemical and histological improvements, such as enhanced spermatogenesis and reduced tissue damage [36]. While all three studies reported antioxidant effects, methodological differences prevented a direct comparison. Asif et al. used chemical antioxidant assays to determine the radical scavenging capacity but do not automatically predict in vivo activity [34]. Both Munir et al. and Ilkhani et al. evaluated the antioxidant potential of the crude hydroethanolic leaf extract of *Epimedium grandiflorum* in animal models, showing an improved endogenous antioxidant defense [35,36]. An important limitation observed across all studies was the lack of standardization and quantification of the main constituents of the used extracts, limiting the reproducibility and the interpretation of the proposed mechanisms. The evidence gathered from the three preclinical studies suggests that *Epimedium grandiflorum* has antioxidant capacities. In order to fill the research gap, this activity requires validation in well-designed clinical studies.

#### 3.2.2. Antiproliferative Activity

Zulfiqar et al. examined the antiproliferative potential of *Epimedium grandiflorum* using in vitro assays [27]. The crude methanolic extract from aerial parts and several isolated prenylated flavonoids were tested using a WST-8 proliferation assay on four cell cancer lines, SK-MEL, KB, BT-549 and SK-OV-3 and two non-cancerous lines, LLC-PK1 and Vero. The methanolic extract at concentrations up to 100 µg/mL produced moderate inhibition of iNOS activity, with an IC_50_ of 70 µg/mL. Isolated compounds exhibited significant antiproliferative effects, with IC_50_ ranging from 9 to 45 µM. These observed antiproliferative effects were associated with the presence of prenylated flavonoids rather than the crude extract [27]. Complementing these findings, Telang et al. evaluated the antiproliferative capacity of an aqueous extract obtained from the leaves and stems of *Epimedium grandiflorum*. The extract was tested in vitro on the MCF-7 cancer cell line, at concentrations of 0.5%, 1.0% and 2.0% (*v*/*v*) [37]. The extract inhibited estrogen-stimulated cell proliferation and induced G1 phase cell arrest, suggesting the potential to suppress the growth of estrogen-dependent tumors [37].

Although the results are promising, the two studies differ considerably in their methodology, including the used extraction solvents, plant parts, concentrations and cell lines. All the factors above can influence biological activity and prevent the rigorous interpretation of the observed results, emphasizing the need for additional studies that would adopt the same methodological strategy, to compare and strengthen the available findings. The evidence for the antiproliferative activity remains limited to in vitro studies, no in vivo or clinical studies are available to further confirm the current findings.

#### 3.2.3. Reproductive and Androgenic Activity

*Epimedium grandiflorum*, also known as horny goat weed, got its common name based on a Chinese folk tale. According to the tale, a goat herder observed an unusually increased libido in his goats after they consumed the plant. *Epimedium grandiflorum* was used in traditional Chinese medicine as an aphrodisiac [38]. Nowadays, these stories are examined by scientists in an attempt to find a rational explanation for their traditional uses. Munir et al. conducted an in vivo study on an *Epimedium grandiflorum* hydroethanolic leaf extract with the purpose of investigating its androgenic effects [35]. The extract was administered orally in doses between 50 and 200 mg/kg to male albino rats with carbon tetrachloride-induced toxicity for 42 days. The treatment significantly increased serum testosterone, luteinizing hormone and follicle-stimulating hormone levels, while decreasing the elevated progesterone and estradiol levels caused by CCl_4_. On a histological level, at the dose of 200 mg/kg, the extract improved testicular weight and restored normal testicular architecture. These androgenic effects may be associated with the presence of prenylated flavonoids, including icariin derivatives, which have been implicated in hormonal regulation [35]. Nasef and El-Sheikh [39] studied the reproductive and androgenic activity of *Epimedium grandiflorum* in vivo on male Sprague–Dawley rats with cadmium chloride-induced infertility. The administration method used was the incorporation of the whole dried plant into the basal diet at 10%, 15% and 20% (*w*/*w*) for 28 days. The supplementation with *Epimedium grandiflorum* resulted in increased serum and testicular testosterone, LH, FSH, GnRH and kisspeptin. The histological analyses revealed an increased weight of accessory reproductive organs and restored testicular architecture in cadmium-exposed rats. The results were associated with the presence of prenylated flavonoids, which may reduce oxidative stress by increasing SOD, CAT and GPx, while decreasing MDA [39]. Another in vivo study conducted by Musa and Obeid [40] evaluated the protective effect of the *Epimedium grandiflorum* aqueous leaf extract on the reproductive function of male albino rats with cisplatin-induced toxicity. The extract was administered alone or co-administered with cisplatin 2 mg/kg, at a dose of 350 mg/kg for a period of 52 days. Cisplatin is an anticancer drug associated with reproductive side effects such us spermatogonial apoptosis, sperm abnormalities and infertility. The extract was able to partially reduce the cisplatin side effects by increasing sperm density and motility and restoring the integrity of the epididymal tissue. The authors associated the observed effects with the presence of prenylated flavonoids that can reduce oxidative stress through up-regulating SOD activity and decreasing lipid peroxidation. The extract’s reproductive capacity is inconclusive. While sperm parameters and histological integrity were partially restored, the study did not evaluate fertility indicators such as mating success or offspring production [40]. The last study that focuses on the protective effects towards male reproductive health of *Epimedium grandiflorum* was conducted by Al-Ibrahimi and Almuhana. Two extracts were orally administered to male albino rats with amlodipine-induced testicular toxicity. Both the aqueous extract at 500 mg/kg and the nano-extract at 1 mg/kg were administered daily, alone or combined with amlodipine at 10 mg/kg, for 45 days. Results showed preserved testicular morphology through maintaining the structure of seminiferous tubule and the integrity of germinal epithelium, counteracting the degenerative changes induced by amlodipine. Although both formulations produced comparable effects, the nano-extract was administered at a much lower dose, which may reflect a higher bioavailability of the extract when it is included in a nano formulation. No direct comparison was made between the two formulations and without quantitative comparative data the superior efficacy of the nano-extract cannot be proven. The study evaluated histological evidence without measuring reproductive hormones or fertility. As a result, the mechanism responsible for the observed reproductive effects of *Epimedium grandiflorum* remains unclear [41].

**Figure 7:**
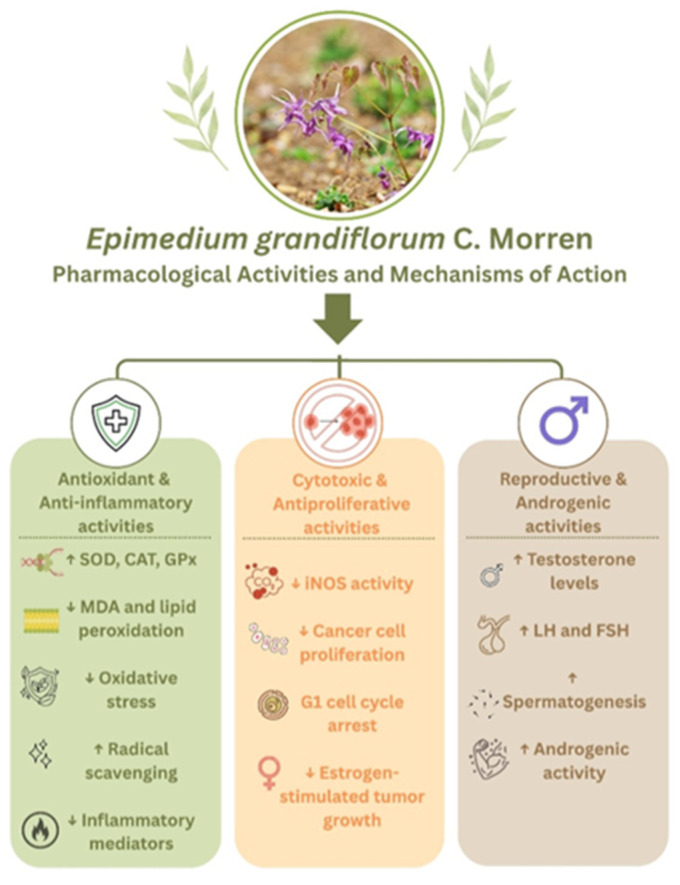
Pharmacological activities and mechanisms of action of *Epimedium grandiflorum* C. Morren.

By analyzing all four studies, *Epimedium grandiflorum* showed consistent protective effects regarding male reproductive function. Among the reviewed studies, Munir et al. [35] and Nasef and El-Sheikh [39] documented an androgenic mechanism that improves hormonal secretion and histological restoration. The next two studies focused on the protective effects rather than androgenic effects. Musa and Obeid [40] presented the partial restoration of sperm quality and epididymal integrity in cisplatin-induced toxicity, while Al-Ibrahimi and Almuhana [41] demonstrated the capacity to preserve testicular morphology in amlodipine-induced toxicity. A substantial gap remains in the current literature, as the lack of standardized methodologies, fertility assessments and molecular mechanism analyses prevent the possibility of formulating a conclusion regarding the therapeutic relevance of *Epimedium grandiflorum* in male reproductive health. Even though the main proposed mechanism centered on the antioxidant capacity of prenylated flavonoids, none of the studies provided a complete characterization of the underlying molecular, functional and hormonal pathways (Figure 7).

## 4. *Epimedium brevicornu* Maxim.

### 4.1. Phytochemistry

Research shows that over 120 compounds are found in *Epimedium brevicornu* Maxim., which can be broadly classified into flavonoids, phenylpropanoids, phenylethanoid glycosides, and lignans. The most notable and widely studied active compounds isolated from the plant are icariin, epimedin A, epimedin B, and epimedin C, icaritin, icariside I and icariside II, baohuoside I (icariside II) and baohuoside VI (Epimedin C), sagittatoside B and epimedoside A from prenylated flavonoids group. Other flavonoids identified in the *Epimedium brevicornu* plant extracts include hyperoside, quercetin-3-O-α-L-rhamnopyranoside, kaempferol-3-O-α-L-rhamnopyranoside, amentoflavone, 2,3-dihydroamentoflavone, bilobetin, apigenin-6,8-di-C-β-D-glucopyranoside, tricin and kumatakenin. Four main phenolic acids namely vanillic acid, isovanillic acid, p-hydroxybenzoic acid and 3,4,5-trimethoxybenzoic acid, and two phenylpropanoids namely, hydroferulic acid and hydrocaffeic acid have been identified. Regarding phenylethanoid glycosides and chromones the major active principles are p-hydroxyphenylethanol, umbelliferone (coumarin), epimedonins (e.g., epimedonins G-K and others). Among the structurally unique compounds identified in *Epimedium brevicornu* are two novel minor flavonoids, breviflavones A and B, which are positional isomers. Notably, only breviflavone B has demonstrated estrogen receptor activity [42,43]. Hong et al., 2009 [44] developed a robust LC-MS/MS method for measuring the two breviflavones in *Epimedium* ethanolic extracts via selected reaction monitoring (m/z 437→m/z 367 for breviflavone A and m/z 437→m/z 351 for breviflavone B) under negative electrospray ionization mode. The authors analyzed the two compounds in ethanolic herbal extracts of the species *E. brevicornu*, *E. koreanum*, *E. pubescens*, *E. sagittatum* and *E. wushanese* and established variations in content ranging from 0.0181 to 0.1791% for breviflavone A and from 0.0026 to 0.0252% for breviflavone B in the plant’s samples [44]. Epimedokoreanin F is a specific flavonoid glycoside possessing an unusual furan ring isolated for the first time from *Epimedium brevicornu* leaf extracts. It revealed a remarkable neuroprotective activity on the model of RSL3-induced ferroptosis in HT22 cells [45]. The four new isoprenylated flavonoid glycosides called ZW1-ZW4 have been extracted from *Epimedium brevicornu* plant material. These specific compounds are potent stimulators of estrogen biosynthesis. ZW1 is a specific inhibitor of phosphodiesterase 5 (PDE5). It is widely used in research to explore the mechanism of flavonoid analogs on estrogen biosynthesis. ZW1 increases estrogen production by upregulating aromatase mRNA and protein expression, promoting the phosphorylation of CREB, and suppressing PDE3 [46]. The three dihydrophenanthrenes (epicornunin C; 4-hydroxy-2,3,6,7-tetramethoxy-9,10-dihydrophenanthrene and Epicornunin D) isolated from the leaves of *Epimedium brevicornu* showed notable cytotoxic effects against HepG2 liver cancer cells, with reported IC_50_ values ranging from 32.8 to 87.3 μM [47].

### 4.2. Biological Activities and Therapeutic Potential

#### 4.2.1. Direct and Indirect Anticancer Activity

The anticancer activity of *Epimedium brevicornu* has been demonstrated through research on its isolated constituents. Network pharmacology and molecular docking were the screening techniques used to identify β-anhydroicaritin and isoliquiritigenin, promising active compounds of *Epimedium brevicornu*. Both compounds were evaluated in vitro using the human triple-negative breast cancer cell line MDA-MB-231 at concentrations of 10 and 20 μmol/L. After treatment, cell viability was suppressed in a dose-dependent manner, with IC50 values of 21.28 µmol/L for β-anhydroicaritin and 23.73 µmol/L for isoliquiritigenin. Cell migration was inhibited after 48 h. Apoptosis and necrosis were induced after 96 h. These effects were attributed to the upregulation of the pro-apoptotic protein Bax and modulation of TBK1/NAK signaling pathway, affecting cell proliferation and immune-inflammatory responses [48]. From a methodological perspective, the reliance on isolated compounds rather than a crude extract, the absence of in vivo validation and the use of a single breast cancer line, limits the ability to determine whether the biological effects are specific to *Epimedium brevicornu*. Dou et al. [49] evaluated the antitumor activity of Icariside II, a prenylated flavonoid isolated from *Epimedium brevicornu*. In vitro, Icariside II was tested on AGS and MGC803 gastric cancer cell lines and on GES-1 normal gastric epithelial cell line, at concentrations of 20, 40 and 60 µM. Icariside II showed no cytotoxicity on GES-1, while inhibiting proliferation and inducing apoptosis of the gastric cancer cell lines. In vivo, Icariside II was administered subcutaneously to BALB/c nude mice at doses of 10, 20 and 40 mg/kg, resulting in dose-dependent inhibition of tumor growth and reduced tumor weight. The proposed mechanism involves the suppression of the Wnt/β-catenin signaling pathway, supported by the downregulation of Wnt3a, β-catenin, C-Myc and Cyclin D1 [49]. An important limitation of this study is the absence of an in vivo toxicity assessment and a comparison with standard chemotherapy. The use of subcutaneous xenograft tumors in immunodeficient nude mice may not fully reflect the complexity of the human tumor microenvironment. Both studies provided evidence of the direct anticancer activity of isolated compounds from *Epimedium brevicornu*. with differences in their methodological approaches. β-anhydroicaritin and isoliquiritigenin exerted antiproliferative effects limited to in vitro testing on a single cancer cell line without in vivo testing. Icariside II provides evidence from both in vitro and in vivo testing, offering stronger support for the proposed mechanism.

In addition to its direct cytotoxic effects, *Epimedium brevicornu* has demonstrated indirect antitumor activity through modulation of the immune system and the tumor microenvironment. An indirect antitumor activity of Icariin, a prenylated flavonoid isolated from *Epimedium brevicornu* was reported by Chen et al. [50]. Tested in vitro on CT26-WT and HCT116 colorectal cancer cell lines co-cultured with M2 macrophages, at concentrations of 10 and 20 µM, Icariin suppressed proliferation, migration and invasion of cancer cells. In vivo, Icariin was orally administered at 100 mg/kg/day to BALB/c mice in an AOM/DSS model with chemically induced colorectal cancer and a CT26-WT syngeneic subcutaneous colorectal tumor model. Treatment resulted in reduced tumor growth and decreased M2 macrophage infiltration. The indirect antitumor effect was mediated through the inhibition of M2 macrophage polarization via the PI3K/AKT signaling pathway [50]. A limitation of this study is the immunodependent mechanism of the observed antitumor effect with no direct cytotoxicity demonstrated. Liu et al. [51] demonstrated an indirect antitumor effect of an *Epimedium brevicornu* extract purchased commercially. The extract was evaluated in vitro at concentrations of 0.25, 0.5 and 1.0 mg/mL against K562 leukemia target cells in a co-culture model with NK-92 effector cells. Results consisted in an enhanced NK-cell mediated cytotoxicity, evidenced by increased production of IFN-γ, granzyme B, perforin and upregulated expression of the NKG2D receptor. In vivo, the administration of the extract in doses of 200, 400 and 800 mg/kg/day to C57BL/6J mice bearing Hepa1-6 hepatocellular tumors resulted in dose-dependent inhibition of the tumor growth, accompanied by reduced tumor weight and increased tumor cells apoptosis. The antitumor effect was mediated through NK-cell activation rather than direct cytotoxicity and attributed to activation of the cGAS-STING signaling pathway, with epimedin C identified as the primary constituent targeting STING [51]. When it comes to indirect anticancer activity, the studies highlight the immunomodulatory potential of *Epimedium brevicornu*, despite differences in experimental standardization and model complexity. Icariin shows immune-mediated anticancer effects through the alteration of macrophage polarization within precise experimental conditions while the other study uses an uncharacterized *Epimedium brevicornu* extract. The incomplete characterization of the extract, which does not include the plant part used, extraction method and solvent, limits study reproducibility and prevents direct comparison with other studies.

#### 4.2.2. Bone-Protective Activity

The osteogenic and anti-osteoporotic activities of a purified polysaccharide extracted from *Epimedium brevicornu* were evaluated in vitro on murine osteoblasts exposed to dexamethasone at 25 and 100 µg/mL. Observed effects included the enhancement of cell survival and osteogenic maturation evidenced by increased proliferation, ALP activity, calcium deposition and reduced apoptosis. These effects were mediated by activation of the PI3K/Akt/mTOR and Wnt/β-catenin pathways associated with the suppression of Bax/Bcl-2/caspase-3 signaling [52]. Using an in vivo model of C57BL/6 female mice with retinoic acid-induced osteoporosis, Ma et al. [53] investigated the anti-osteoporotic activity of an Epimedium brevicornu leaf aqueous extract. The extract was administered orally at 0.81 g/kg/day, and resulted in increased femur length, elevated serum IGF-1 and reduced CTX-I via the modulation of gut-bone axis and the inhibition of NLRP3/caspase-1/IL-1β inflammatory signaling pathway [53]. Another aqueous extract from *Epimedium brevicornu* leaves, was tested by Liu et al. [54] for anti-osteoporotic properties. Female Sprague–Dawley rats with ovariectomy-induced osteoporosis received the extract orally at 0.27, 0.81 and 2.43 g/kg/day. The reported effects included restored bone mineral density and increased serum levels of estradiol, osteoprotegenin and bone Gla protein through the modulation of NPY, CGRP/CRLR, VIP and substance P pathways [54]. A recent study conducted by Cai et al. [55] investigated the effects of *Epimedium brevicornu* nanovesicles on female C57BL/6 ovariectomized mice. The extracellular nanovesicles were administered orally at 1 × 10^9^ particles/kg/day. The restoration of bone density and microarchitecture was caused by stimulating angiogenesis through the activation of VEGF/VEGFR2 signaling pathway [55].

The identified studies that investigated the bone-protective and anti-osteoporotic activity of *Epimedium brevicornu* used different types of formulations, ranging from purified isolated polysaccharides to crude aqueous extracts and extracellular nanovesicles leading to different proposed mechanisms of action. The observed beneficial effects were consistent across multiple osteoporosis models, implying that the anti-osteoporotic activity of *Epimedium brevicornu* is not limited to a single pathological pathway. Despite the favorable preclinical evidence, no clinical studies were done to validate them and confirm their translational relevance to humans.

#### 4.2.3. Neuroprotective Effects

A freeze-dried hydroethanolic extract containing 57% flavonoids from *Epimedium brevicornu* leaves was evaluated by Li et al. [56] for its neuroprotective and anti-neuroinflammatory activities. The study used both in vitro and in vivo assessments. In vitro, the extract was tested at 20–40 µg/mL on a BV2 microglial cell line stimulated with LPS. The model used for the in vivo testing was C57BL/6 male mice with LPS-induced cognitive impairment who received the extract via oral administration in doses of 50–200 mg/kg/day. The extract ameliorated cognitive deficits, suppressed microglial and astrocytic activation, and reduced Aβ42 deposition through inhibiting PI3K/AKT and cGAS-STING signaling pathways [56]. Continuing to investigate the neuroprotective potential of *Epimedium brevicornu*, Niu et al. [57] analyzed a standardized flavonoid extract, administered orally, at 50, 100 and 200 mg/kg/day to male Sprague–Dawley rats with chronic cerebral hypoperfusion induced by 2VO. The treatment ameliorated spatial learning and memory impairments, alleviated white matter demyelination and restored mature oligodendrocytes population through inhibiting the Lingo-1/Fyn/ROCK axis and activating the BDNF/TrkB, NRG-1/ErbB4 and PI3K/AKT/CREB pathways [57].

Despite addressing different neuropathological contexts, both studies support the neuroprotective potential of *Epimedium brevicornu* flavonoid extracts through different yet partially overlapping mechanisms. Li et al. [56] adopted a dual in vitro/in vivo design, while Niu et al. [57] relied on an in vivo model that closely resembled human vascular cognitive impairment. Both studies implicate the PI3K/AKT pathway, suggesting a mechanistic overlap, although each identified distinct signaling targets. The primary limitations discovered by analyzing these studies are using only male mice as in vivo models and the absence of any clinical data.

#### 4.2.4. Androgenic and Reproductive Enhancing Activities

Wang et al. conducted a study that focused on the ethanol extracts of raw and suet leaves of *Epimedium brevicornu* and their erectile-enhancing effects. The extracts were administered orally at 2.25 g/kg/day to C57BL/6 male mice with kidney-yang deficiency induced by corticosterone. Results showed elevated serum testosterone, restored CRH and ACTH hormone levels through systemic regulation of the neuroendocrine-immune network and local improvements caused by eNOS upregulation and PDE5A inhibition [58]. A recent study shifted the focus from an *Epimedium brevicornu* extract to investigating an isolated compound, more precisely icariin. Liao et al. used a C18-4 mouse spermatogonial stem cell line and C57BL/6 male mice with reproductive damage caused by H_2_O_2_. In vitro, the used concentrations were between 2.5 and 15 µmol/L and for the in vivo testing, icariin was administered intraperitoneally at 100 mg/kg/day. Icariin promoted stem cell proliferation, reduced sperm abnormalities and prevented oxidative DNA damage through PDE5A inhibition [59]. Even if both studies supported reproductive-enhancing activities they have different methodological approaches, Wang et al. tested a crude extract showing systemic hormonal and vascular effects but without isolating any active compounds, limiting mechanistic clarity while better reflecting whole-plant activity. On the other side, Liao et al. focused on icariin to identify the PDE5 inhibition as a more specific mechanism of action. Taken together, they imply that the effects of the *Epimedium brevicornu* extracts may be determined by icariin.

## 5. *Epimedium sagittatum* (Siebold & Zucc.) Maxim.

### 5.1. Phytochemistry

Phytochemical research has identified over 130 metabolites in *Epimedium sagittatum* (Sieb. et Zucc.) Maxim. including the heavily studied prenylated flavonoids like icariin and epimedin A-C. The other primary bioactive compounds of the group responsible for the herb’s traditional uses are icariside I, icariside II, icaritin, sagittatoside A, sagittatoside B, sagittatoside C, baohuoside I, baohuoside II, desmethylicaritin, desmethylanhydroicaritin, breviflavone A, breviflavone B, ikarisoside A, ikarisoside B, ikarisoside C, epimedokoreanin B and epimedoside A. The other common flavonoids for the species are genistein, daidzein, luteolin, hyperoside, apigenin, chrysoeriol and tricin. The most abundant phenolic compounds found in the plant are salidroside, protocatechuic acid, p-hydroxybenzoic acid, p-dihydro coumaric acid, trans-p-coumaric acid, chlorogenic acid. Lignans ikarisoside E6, ikarisoside E7, icariol A1, icariol A2, 3,5-demethoxy-syringaresinol-glucoside, (−)-olivil, EL1, EL2, EL9 and (+)-dihydro-dehydrodiconiferyl alcohol-4-O-β-D-glucopyranoside were identified in *Epimedium sagittatum* as a characteristic substance from the group [60,61]. The other compounds reported in Epimedium sagittatum are ionones and their derivatives such as ikarisoside B2, ikarisoside B8 and ikarisoside B9 [60]; phenethyl alcohol glycosides such as ikarisoside D2 and ikarisoside D3 [60,62]; phytosterols (β-Sitosterol); and alkaloids (e.g., Magnoline, particularly concentrated in stems/rhizomes). While many prenylated flavonoids are widely distributed across the *Epimedium genus*, Epimesatines (A–S) are unique prenylated flavonoids that have been isolated specifically from aerial parts of *Epimedium sagittatum*. Epimesatines A–I are nine compounds known for displaying strong inhibitory activities against sphingosine kinase 1 (SPHK1), which is linked to anti-lung cancer pathways [63]. Epimesatines P–S are four recently isolated cytotoxic flavonoids with unique ring structures. Characteristic features of epimesatines Q and R is the presence of furan skeleton. Epimesatines P–S had notable cytotoxic effect on the against MCF-7 human breast cancer cells. Epimesatins Q and R display significantly better activity against MCF-7 cell line than epimesatins P and S, suggesting that the presence of a furan moiety may increase their action against MCF-7 cell line. Epimesatin Q showed stronger inhibitory activity at 1.27 μM compared to the positive control, docetaxel with an IC50 of 2.13 μM, revealing its healing potential in a breast cancer treatment. Moreover, none of the tested substances exhibited obvious toxicity toward MCF-10A human breast epithelial cell line [64]. Three unique prenylflavonol glycosides called sagittasine A, B, and C have been structurally defined from the n-BuOH extract of the aerial parts of *Epimedium sagittatum* [61]. The other two specific acylated flavonol glycosides kaempferol-3-O-(2″-E-p-coumaroyl,4″-Z-p-coumaroyl)-alpha-l-rhamnopyranoside and kaempferol-3-O-(3″-Z-p-coumaroyl,4″-E-p-coumaroyl)-alpha-l-rhamnopyranoside have also been described in n-BuOH extract from the aerial parts of *Epimedium sagittatum* [61]. Five new prenylflavones yinyanghuo A, yinyanghuo B, yinyanghuo C, yinyanghuo D and yinyanghuo E, were isolated from the leaves of *Epimedium sagittatum*. Compounds yinyanghuo A and yinyanghuo B showed significant antiplatelets induced by arachidonic acid [65]. A novel tetraazacyclododecane alkaloid, 2-acetyl-1,4,7,10-tetraazacyclododecane, was isolated from the stems of *Epimedium sagittatum*. The structure of the new tetraazacyclododecane was determined by the interpretation of the spectroscopic data [66]. A new β-ionone has been described for the first time from the stems of *Epimedium sagittatum* (Berberidaceae) [67].

### 5.2. Biological Activities and Therapeutic Potential

#### 5.2.1. Anti-Inflammatory Effects

The anti-inflammatory activity of an ethyl acetate extract from *Epimedium sagittatum* aerial parts, was investigated in vitro by Yan et al. After characterizing the extract using HPLC-MS/MS, eight compounds were identified, with the most abundant being Icariside II and Icariin. The extract was tested at 12.5–50 µg/mL on RAW264.7 macrophages stimulated with LPS. The effects presented were reduced TNF-α and IL-2 production and a dose-dependent suppressed NO synthesis. The potentially involved mechanisms consisted of the inhibition of TLR4/MD-2 complex formation, a decrease in NF-κB phosphorylation and the prevention of p65 nuclear translocation [68]. A more recent study conducted by Wang et al. tested a preparation obtained from the aerial parts of *Epimedium sagittatum* for its anti-inflammatory potential and compared it with icariin. Icariin was identified as the main component of the tested preparation through HPLC characterization. The preparation was orally administered to male Sprague–Dawley rats at 1.17 and 4.68 g/kg/day for 4 weeks, while icariin was tested in vitro at 0.5–50 µM on NRK-52e renal epithelial cells induced with Adriamycin. The treatment with *Epimedium sagittatum* improved renal histopathology, reduced inflammatory markers such as IL-1β, TNF-α, IL-6 and MCP-1 and attenuated oxidative stress, while icariin reproduced these effects in vitro. The mechanism responsible for these effects is the activation of PI3K/AKT signaling pathway followed by the downstream antioxidant response of Nrf2/HO-1/NQO-1 [69].

Analysis of both studies confirms the anti-inflammatory potential of *Epimedium sagittatum* via different mechanisms, TLR4/MD-2/NF-κB signaling pathway for the in vitro study conducted by Yan et al. and PI3K/AKT activation in the in vivo study conducted by Wang et al. Through combining *Epimedium sagittatum* in vivo testing with in vivo icariin testing, Wang et al. provided stronger translational evidence compared with Yan et al. who remained limited to a single cell-based model without any in vivo validation. Neither study fully specifies extract standardization criteria, limiting reproducibility and direct-cross studies comparison.

#### 5.2.2. Neuroprotective Effects

In order to investigate the possibility of using *Epimedium sagittatum* as a source for neuroprotective therapeutic agents, Sheng et al. used Icariin, a prenylated flavonoid found in high concentration in this species. Icariin was orally administered at 30, 60 and 120 mg/kg/day to male Sprague–Dawley with Alzheimer’s disease, activating BDNF/TrkB/Akt/CREB signaling cascade, which appeared to counteract synaptic deterioration and memory impairment induced by the Aβ_1-42_. Icariin restored cognitive performance in a dose-dependent matter, increasing synapse density and improving postsynaptic density thickness [70]. Shifting the focus towards Parkinson’s disease, Chen et al. studied the neuroprotective effects of purified icariin isolated from *Epimedium sagittatum*. In vitro, icariin was tested at 0.01 µM on MES23.5 dopaminergic cell lines, while the in vivo model consisted in C57BL/6 ovariectomized female mice exposed to MPTP, who received icariin intragastrically at 50, 100 and 200 mg/kg/day. The treatment preserved striatal dopamine levels and reduced the number of tyrosine hydroxylase-positive neurons affected by apoptosis. The neuroprotective mechanism consists of the activation of PI3K/Akt and MEK/ERK intracellular pathways, which results in an increased Bcl-2 and a decreased Bax and caspase-3 that will alter the apoptotic balance. Another investigation conducted by Wu et al. used Sprague–Dawley male rats with Parkinson’s disease induced by rotenone, to explore the potential neuroprotective effects of icaritin, a derivative of icariin. Icaritin was orally administered in doses of 3.27, 6.57 and 13.08 mg/kg/day. Single cell RNA sequencing identified a new synapse-rich cell cluster (SRC) as being the target of the icaritin treatment. Icaritin is binding to transcription factors such as SMAD3, CEBPB and NR3C2 within these SRCs, as well as normalizing astrocyte-mediated energy and phospholipid metabolism via GPI-PLD modulation. The reported effects of icaritin are motor function restoration and dopaminergic loss prevention [71].

After a critical analysis of all three studies, a major advantage across all of them is the use of isolated and purified compounds such as icariin and icaritin, reducing the ambiguity of the pharmacological effects of crude plant extracts. Despite their shared foundation, the scientific principles behind their experimental designs show significant variation. While the study conducted by Chen et al. demonstrates a higher level of scientific rigor by integrating both in vitro and in vivo tests, Wu et al. uses advanced single-cell RNA sequencing to reveal new cellular targets like SCRs, and Sheng et al. relies exclusively on an observational in vivo approach without pharmacological intervention to validate the proposed mechanism. Finally, all three studies use in vivo models with acute chemically induced injuries that do not entirely replicate chronic, progressive human neurodegeneration.

#### 5.2.3. Erectile-Enhancing Activity

Li et al. examined the erectile-enhancing potential of three purified 8-isopentenyl flavonoids, more precisely of icariin, 2-O″-rhamnosylicaridide II and baohuoside I, extracted from *Epimedium sagittatum*. The test system incorporated both an in vitro enzymatic assay and CCSMC rat corpus cavernosum smooth muscle cells, all three isolated compounds were tested at 10 µM causing elevated intracellular cGMP levels and reduced calcium influx. The cellular mechanism controlling smooth muscle relaxation involves the direct inhibition of PDE5A1, which activates protein kinase G downstream, reducing cytosolic Ca^2+^ and facilitating cavernosal vasodilatation [72]. The potential PDE5A inhibition caused by purified flavonoids isolated from a hydroethanolic extract of *Epimedium sagittatum* leaves, was examined in an in vitro study conducted by Fang et al. The compounds were tested at different concentrations using an in vitro enzymatic system combined with X-ray crystallography, 0.1593–25 µM for the surface plasmon resonance binding assay and 5 mM for the crystallographic soaking drops. Crystallographic analysis confirmed that these molecules bind to the PDE5A catalytic domain [73]. Further investigation demonstrated that prenylhydroquinones and flavonoids, including sagittatoside I, isolated from a hydroethanolic extract of *Epimedium sagittatum* leaves, exhibit erectile enhancement activity. The compound was tested combining an in vitro enzymatic analysis with molecular docking throughout a 7-concentration series. Sagittatoside I exhibited potent PDE5A inhibition with an IC_50_ of 1.86 µM. Biding affinity measurements above −7.0 kcal/mol confirmed stable, targeted binding directly within the PDE5A active site, validating sagittatoside I as a major contributor to the aphrodisiac activity of *Epimedium sagittatum* [74]. A crude hydroethanolic extract made from *Epimedium sagittatum* leaves was evaluated by Zheng et al. for its aphrodisiac properties. The C57BL/6 male mice with kidney-yang deficiency induced by corticosterone administration, received daily doses of 1.13 and 3.39 g/kg for 21 days. After the treatment, testosterone serum levels were elevated, and endothelial nitric oxide synthase gene in corpus cavernosum was enhanced through direct suppression of PDE5A activity in order to ameliorate sexual dysfunction [75].

All four investigations reveal methodological differences as well as extensive, multilayered validation of *Epimedium sagittatum* aphrodisiac activity. The enzymatic-based evaluations used purified, isolated flavonoids from *Epimedium sagittatum* extracts, which led to successfully elucidating the exact cellular mechanisms of PDE5A inhibition. The weakness of these studies was their strict reliance on in vitro and in silico techniques, lacking the physiological parameters necessary to confirm bioavailability and tissue penetration. In contrast, the in vivo study provides the vital physiological insight that is missing in these biochemical studies. Any future preclinical studies must combine these approaches, utilizing flavonoids isolated from *Epimedium sagittatum* extracts within standardized in vivo models in order to validate their clinical potential for enhancing erectile function.

## 6. *Epimedium koreanum* Nakai

### 6.1. Phytochemistry

Phytochemical research on *Epimedium koreanum* Nakai. has identified numerous bioactive secondary metabolites, primarily consisting of flavonoids, as well as lignans, phenols, and alkaloids. Li et al., 2016 [76] isolated, for the first time, three novel acylated prenylflavonol glycosides, namely korepimeosides A-C along with twenty known derivatives (epimedokoreanosid I, caohuoside B, korepimedoside C, epimedokoreanosid II, korepimedoside A, koreanoside E, 3″′-carbonyl-2″-β-L-quinovosyl icariside II, hexandraside F, epimedin B, icariin II, icariin, sagittatoside, 2″-O-rhamnosyl icarisoside II, sagittatoside B, anhydroicaritin-3-O-b-D-fucopyranosyl(1→2)-α-L-rhamnopyranoside, icarisoside B, 2″-O-rhamnosyl ikarisoside A, icarisoside F, icarisoside A, and epimedoside C) from the EtOAc extract of the aerial parts of *Epimedium koreanum*. The structures of three new compounds were established on the basis of chemical and spectroscopic methods as icaritin 3-O-[2,6-O-diacetyl-b-D-glucopyranosyl(1→3)-4-Oacetyl-a-L-rhamnopyranoside], icaritin 3-O-[3,6-O-diacetyl-b-D-glucopyranosyl(1→3)-4-O-acetyl-a-L-rhamnopyranoside], and icaritin 3-O-[3,-O-acetyl-b-D-glucopyranosyl(1→3)-4-O-acetyl-a-Lrhamnopyranoside], 7-O-b-D glucopyranoside [76]. Su et al. conducted a study of the chemical composition of the aerial parts of *Epimedium koreanum* and determined the chemotaxonomic significance of all 21 isolated compounds. They described the isolation of 11 flavonoids divided into three groups, anhydroicaritin (korepimedoside C, epimedokoreanoside I, epimedin B, icariine and icariside II), desmethylanhydroicaritin (epimedoside E, epimedoside A, hexandraside E and ikarisoside B) and quercetin glycoside (quercetin 3-O-rhamnoside and hyperoside), and defined anhydroicaritin and desmethylanhydroicaritin with the pentenyl substituent at C-8 position as the most characteristic substances of the genus *Epimedium*. As for the other compounds, epimedin B, icariine and icariside II have been applied as reference standards in the quality control and pharmaceutical analysis of several species in the genus *Epimedium*, such as *E. koreanum*, *E. brevicornum* and *E. pubescens*. The other broadly distributed desmethylanhydroicaritin flavonoids in the genus *Epimedium* are epimedoside E, epimedoside A, hexandraside E and ikarisoside B. The anhydroicaritin derivatives with acetylated sugar groups called korepimedoside C and epimedokoreanoside I, have been determined as distinctive components of *E. koreanum*. Generally, the various identified prenylflavonoids serve as chemotaxonomic markers for *E. koreanum*. Regarding the four isolated lignans, (–)-olivil and lariresinol are further classified as furan types, while (+)-cycloolivil (8) has a carbocyclic ring. Fourth compound is a neolignan glycoside with two phenylpropanoids linked by an 8-O-4′ bond. (–)-Olivil and neolignan glycoside were reported only in *E. sagittatum* and *E. grandiflorum* before. (+)-Cycloolivil is a major lignin that has been extracted from many species in the genus. Lariresinol has been isolated from the family *Berberidaceae* for the first time. The six main phenolics (4-hydroxybenzoic acid, protocatechuic acid, naringeninic acid, caffeic acid, methyl chlorogenate) have been determined in *E. koreanum*, of which protocatechuic acid, naringeninic acid and caffeic acid have not been previously reported for *E. koreanum*. Protocatechuic acid and naringeninic acid have been proved in *E. sagittatum*, while caffeic acid has been identified in *E. brevicornum*. Methyl chlorogenate has been isolated from family Berberidaceae for the first time. The dihydrophenanthrene derivative epimedoicarisoside A is a specific component only for *E. koreanum* and has chemotaxonomic significance for the species. Zhang et al., 2020 discovered two new flavonol glycosides, koreanoside F and koreanoside G, in the leaves of *Epimedium koreanum* [77]. The key aporphine-type alkaloids found in the leaves of E. koreanum are epimediphine and magnoflorine. Epimediphine acts as a dose-dependent inhibitor of acetylcholinesterase (AChE), while magnoflorine, present in various *Epimedium* species, has been known for its immune-regulating, antioxidant, and mild hypotensive effects [78].

### 6.2. Biological Activities and Therapeutic Potential

#### 6.2.1. Anticancer Activity

Lee et al. conducted an in vitro study using three human brain cancer cell lines, A172, U373MG and T98G, with the purpose of evaluating the anticancer activity of a crude aqueous *Epimedium koreanum* extract. The extract was applied at a concentration of 200 µg/mL. The effects were observed only for A172 cells and included the suppression of cancer cell migration and invasion. *Epimedium koreanum* exerted its antitumor effects by downregulating NF-κB nuclear translocation which further reduced MMP-9, blocking the degradation of the extracellular matrix [79]. From an *Epimedium koreanum* extract to compounds isolated from this species, Epimedokoreanin C was investigated by Liu et al. in an in vitro study on NCI-H292 and A549 human lung cancer cell lines. The compound was used at concentrations ranging from 5 µM to 25 µM. Post-treatment effects included reduced cell viability and migration, as well as increased accumulation of cytoplasmatic vacuoles. Epimedokoreanin C triggered abnormal micropinocytosis by dysregulating the Rac1 and Arf6 proteins, promoting intracellular fluid accumulation, while inhibiting PIKfyve enzyme to prevent the breaking of the fluid vacuoles [80]. Another compound isolated from *Epimedium koreanum* is Epimedokoreanin B. This phytoconstituent was investigated by Zheng et al. for its antitumor potential both in vitro and in vivo. In vitro, the compound was tested in A549 and NCI-H292, two human non-small cell lung cancer lines, at 5–40 µM. In vivo, the tumor-bearing zebra fish embryos were exposed to liquid environments containing Epimedokoreanin B at 10 µM and 20 µM. Epimedokoreanin B induces paraptosis by triggering endoplasmic reticulum stress and blocking autolysosome formation, which leads to the inhibition of cell proliferation and migration [81]. The crude extract stabilized the cellular microenvironment by suppressing NF-κB-mediated cell invasion, the isolated prenylated flavonoids destroy apoptosis-resistant cancer cells by triggering distinct non-apoptotic death pathways. Unfortunately, all three studies failed to identify the direct molecular binding sites that are responsible for initiating these pathways. Collectively, these studies highlight an important knowledge gap. Because the crude extract and the isolated compounds were investigated independently, it remains unclear how these constituents interact within the natural phytochemical matrix. To bridge this gap, future studies should evaluate the crude extract in parallel with different combinations of these isolated compounds using in vivo models. These studies could determine whether their distinct mechanisms interact synergistically to amplify tumor suppression or antagonistically to limit therapeutic efficacy.

#### 6.2.2. Antioxidant Potential

The antioxidant activity of a crude hydroethanolic extract from *Epimedium koreanum* leaves was studied by Zhao et al. through an in vitro DPPH chemical assay. The observed effects revealed that the sample with the highest radical scavenging capacity had an IC_50_ value of 87.44 µg/mL and the sample with the lowest activity had an IC_50_ value of 118.12 µg/mL. Antioxidant activity occurs through the phenolic groups of the preserved flavonoids, which facilitate electron delocalization and hydrogen bounding in order to neutralize free radicals [82]. The second study that investigated the antioxidant potential was conducted by Li et al. This investigation focused on a purified polysaccharide fraction isolated from *Epimedium koreanum* called EFPN-1 that was tested using vitro chemical assays. For the hydroxyl radical and ferrous ion chelation tests, EFPN-1 was applied at doses ranging from 1 to 8 mg/mL, and for the DPPH tests the doses ranged from 0.05 to 0.4 mg/mL. The maximum concentration had a 90.05% hydroxyl radical scavenging rate and a strong iron chelation capacity. EFPN-1 acts as an electron donor by stabilizing free radicals and as an iron chelator to block the production additional oxidative stress [83]. Both studies contain a comprehensive analytical characterization of the tested materials through chromatography and spectrometry, linking phytoconstituents to their bioactivities. On the other side, both investigations rely exclusively on in vitro chemical tests to evaluate antioxidant activity. The absence of any in vitro, in vivo or clinical evidence limits the physiological relevance of the studied agents, thereby leaving their bioavailability and metabolic properties unverified.

#### 6.2.3. Cardioprotective

Irfan et al. studied the antiplatelet and antithrombotic properties of an ethyl acetate fraction obtained from *Epimedium koreanum*. Washed human and rat platelets were used for the in vitro assays and the fraction was applied at concentrations ranging from 15.6 to 200 µg/mL. For the in vivo tests, the *Epimedium koreanum* fraction was orally administered at 100 and 300 mg/kg to AV-shunt rats, and intraperitoneally at 300 mg/kg to mice with tail bleeding injury. The observed effects included the inhibition of platelet aggregation, reduced thrombus weight and prolonged bleeding time. The mechanism of action operates by suppressing intracellular calcium release and ATP secretion while inhibiting integrin αIIbβ3 activation [84]. Kim and Shim continued to further investigate the cardioprotective potential of the *Epimedium koreanum* by focusing on atherosclerosis prevention. A hydroethanolic crude extract of *Epimedium koreanum* and eight of its isolated flavonoids were tested in vitro on lipoproteins derived from human plasma, the extract was applied at 10 and 100 µg/mL, while the isolated flavonoids were applied up to 40 µM. Treatments preserved the structural integrity and anti-atherogenic function of HDL by preventing apoA-I aggregation [85]. Both studies trace the studied therapeutic activities back to specific flavonoids, strengthening the pharmacological profile of *Epimedium koreanum*. Irfan et al. links antithrombotic activity to icariin, while *Kim and Shim* attributed HDL preservation to des-O-methyl-β-anhydroicaritin. The gaps identified in these studies referred especially to methodological approaches; Kim and Shim relied only on in vitro testing, while Irfan et al. incorporated in vivo experiments as well. However, because the extract was administered in different ways to different models, it produces a pharmacokinetic inconsistency that complicates the understanding of the extract’s gastrointestinal absorption.

#### 6.2.4. Neuroprotective

The evaluation of the neuroprotective activity of *Epimedium koreanum* was performed by Wu et al. The total flavonoid fraction derived from the dried aerial parts of *Epimedium koreanum* was tested on MPP+ exposed MES23.5 dopaminergic cell line at 0.125, 0.25 and 0.5 mg/mL. The same fraction was orally administered to C57BL/6 mice with injuries induced by MPTP at 25, 50 and 100 mg/kg. Treatment prevented striatal dopamine loss, protected tyrosine hydroxylase-positive neurons and maintained cellular viability through an anti-apoptotic mechanism that regulates the levels of Bcl-2 and Bax [86]. Jeong et al. evaluated a hydromethanolic subfraction of *Epimedium koreanum* and magnoflorine, an isolated compound. In vitro testing involved an enzymatic assay; the concentrations used went up to 200 µg/mL for the subfraction and up to 20 µg/mL for magnoflorine. Results of the in vitro tests showed that both the hydromethanolic subfraction and magnoflorine inhibited the AChE enzyme in a concentration-dependent matter. For the in vivo experiments, an ICR mouse model with induced cognitive impairment by Aβ_1-42_ intracerebroventricular injection received the subfraction in doses of 200 and 300 mg/kg through oral administration. The *Epimedium koreanum* subfraction reversed spatial and memory deficits by restoring the cholinergic function [87]. The first study used an acute mode with MPTP-induced injuries, which does not fully reflect the progressive nature of human neurodegeneration. The second study used in vitro testing only for the hydromethanolic subfraction, leaving the systemic efficacy of magnoflorine unconfirmed. As a future direction, research should incorporate chronic disease models that would better mimic human neurodegeneration and compare whole extracts to isolated compounds for a better understanding of the possible mechanisms.

## 7. *Epimedium pubescens* Maxim.

### 7.1. Phytochemistry

The principal bioactive marker compounds isolated from *E. pubescens* Maxim. include flavonoid glycosides—icariin, epimedin A, epimedin B, and epimedin C. These are the primary quality control markers designated by the Chinese Pharmacopeia. Other major prenylated flavonols include icaritin, anhydroicaritin, desmethylanhydroicaritin, baohuoside I, baohuoside II (icariside II), icariside I, epimedokoreanin B. The major flavonoids and phenolic compounds identified in the plant include tricin, kaempferol, daidzein, and 4-hydroxyethyl benzoate. Phenolic acids (such as chlorogenic acid) and alkaloids (such as magnoflorine) are additional constituents of the phytochemical profile of *Epimedium pubescens* Maxim. A recent publication presented a non-targeted metabolomics study analyzing metabolic changes in *E. pubescens* under light stress. A total of 476 differential metabolites have been detected, with flavonoids displaying the most prominent upregulation (17.86%), including 6-hydroxy luteolin, pretenol A, and catechin derivatives. KEGG enrichment analysis showed that pathways such as phenylalanine metabolism, flavone, and flavonol biosynthesis were significantly enriched, highlighting the role of secondary metabolite accumulation in plant adaptation to light stress. The results provide theoretical insights for optimizing cultivation practices and improving the medicinal properties of *E. pubescens* [88]. Wang et al., 2020 [89] developed an efficient method for the extraction of main biologically active components epimedin A, epimedin B, epimedin C and icariin from *Epimedium pubescens* using deep eutectic solvents (DESs). DES composed of lactic acid and choline chloride with the ratio of 2:1 was selected as the most promising. The maximum extraction yields of 98%, 99%, 97%, 96% for epimedin A, epimedin B, epimedin C and icariin, respectively, were obtained using a water content of 17.5% (*v*/*v*), the volume of DES aqueous solution of 3.14 mL, and extraction time of 21 min [89].

### 7.2. Biological Activity and Therapeutic Potential

#### Anti-Osteoporotic Activity

Recent research has focused on the osteogenic activity of *Epimedium pubescens* by investigating the major isolated prenylated flavonoid, icariin, on BMSCs human bone mesenchymal stem cells. Icariin was used in concentrations ranging from 1 to 10 µM, at 5 µM icariin showed the strongest cell proliferation and osteogenic differentiation. The bone-restorative effects were driven by the upregulation of GLI-1 [90]. Another in vivo study investigated chondroprotective and osteogenic activities of icariin, the major bioactive compound isolated from *Epimedium pubescens* in vivo, on chickens with thiram-induced tibial dyschondroplasia. Icariin was administered orally daily into the bird’s drinking water at 10 mg/kg. The condition of the affected tibial bones showed an improvement through the restoration of normal growth plate structures and enhanced angiogenesis. Icariin manifested its effects by increasing the expression of BMP-2, which stimulates bone formation and chondrocyte differentiation [91]. Exploring the anti-osteoporotic activity of the same isolated compound, Si et al. used a dual test system featuring both an in vitro RAW264.7 cell line and an in vivo ovariectomized rat model. The cell culture was treated with icariin at 2.5, 5 and 10 µM, while the rats received icariin via oral administration at 125, 250 and 500 mg/kg. The treatment produced osteoclastogenesis inhibition and in vitro results showed a marked suppression of osteoclast differentiation genes such as NFATc1, Ctsk and trap; in vivo, the model had an improved bone density and reduced trabecular bone loss. The osteoprotective effects were achieved through the modulation of the Cullin 3/Nrf2/OH signaling pathway [92]. These studies establish icariin, the key prenylated flavonoid of *Epimedium pubescens,* as a possible anti-osteoporotic agent. Using the same compound across all studies offers the possibility to understand how icariin acts in multiple in vivo and in vitro models. This targeted approach highlights icariin’s ability to stimulate bone formation while inhibiting bone resorption. In the future, new research must prioritize controlled clinical trials to validate the therapeutic potential of *Epimedium pubescens* extracts and bioactive compounds.

## 8. Comparative Synthesis Across *Epimedium* Species

A comparative analysis of the five *Epimedium* species revealed a shared chemical framework, variations in their mechanisms of action and different quality of supporting evidence, suggesting that these species are not interchangeable.

### 8.1. Pharmacological Activities, Bioactive Compounds and Evidence Levels

The pharmacological evidence across the reviewed *Epimedium* species reveals common patterns and significant gaps in the current literature. An imbalance in research coverage is evidenced by the high number of studies focusing on *Epimedium brevicornu* and *Epimedium sagittatum* biological activities, all supported by in vitro and in vivo data. In contrast, Epimedium pubescens is represented only by the anti-osteoporotic activity, investigated through the isolated compound icariin. This difference reflects a publication bias, as species like *Epimedium brevicornu* and *Epimedium sagittatum* have been prioritized in research following the Chinese Pharmacopeia, leaving species like *Epimedium pubescens* and *Epimedium grandiflorum* understudied.

A systematic weakness across all reviewed species is represented by the lack of clinical validation. All documented biological effects are based exclusively on preclinical models. For species like *Epimedium grandiflorum* and *Epimedium koreanum*, most of their pharmacological activities are limited to in vitro validation; while they provide insights into the underlying mechanisms, they are unable to predict bioavailability or metabolic stability in vivo. The antioxidant profile for both species relies on basic chemical assays or single-enzyme tests, which prevents assessing the true physiological impact without the support of in vivo data. The bone-protective and anti-osteoporotic activities of *Epimedium brevicornu* benefit from consistent in vivo validation, positioning it as a strong candidate for future translational research.

Another limitation is the heterogeneity of the bioactive agents tested across the included studies. Several biological activities have been attributed to crude or partially characterized extracts, NK cell activation for *Epimedium brevicornu* and the androgenic effects for *Epimedium grandiflorum*, where the specific phytochemical constituents responsible for these effects were not fully identified and extract standardization was not reported. This limits mechanistic interpretation and hinders direct comparisons across *Epimedium* species. By comparison, the neuroprotective and bone-protective activities of *Epimedium brevicornu* and *Epimedium sagittatum* are supported by both in vitro and in vivo evidence, but the findings are based on partially characterized flavonoid fractions rather than fully defined bioactive compounds. The anti-osteoporotic activity of *Epimedium pubescens* is supported by the consistent use of isolated icariin, providing greater reproducibility and stronger compound-specific activity. These differences are more likely to reflect variability in methodological rigor and phytochemical characterization across the reviewed literature rather than genuine differences in the therapeutic potential of the species (Table 2).

### 8.2. Signaling Pathways and Mechanisms of Action

The PI3K/AKT axis is the most conserved pathway within the genus, reported in *Epimedium brevicornu*, *Epimedium sagittatum* and *Epimedium koreanum* across pharmacologically distinct contexts, such as immunomodulation, neuroprotection and anti-inflammatory signaling. This suggests that PI3K/AKT modulation is not specific to a certain species, but rather a genus mechanistic tendency associated with the shared prenylated flavonoids, especially icariin and icaritin.

NF-κB inhibition, Bcl-2/Bax regulation and eNOS upregulation are shared across two or three species, yet they operate by different mechanisms depending on the biological context. NF-κB inhibition has been reported in the anti-inflammatory activity of *Epimedium grandiflorum* and *Epimedium sagittatum*, and in the anticancer activity of *Epimedium koreanum.* The variability reflects the pleiotropic nature of NF-κB signaling and suggests that shared pathway involvement should not be interpreted as evidence of equivalent pharmacological effects. Modulation of the Bcl-2/Bax axis is associated with pro-apoptotic effects in cancer models for *Epimedium brevicornu* and *Epimedium sagittatum*, and neuroprotective effects of *Epimedium koreanum.* The same molecular pathway can lead to opposite functional outcomes, highlighting that cellular context and extract concentration are as important as pathway identity in determining pharmacological effects. By comparison, the shared eNOS/PDE5A axis in *Epimedium brevicornu* and *Epimedium sagittatum* represents a consistent mechanism, both species contain icariin as a major constituent, and their erectile-enhancement effects converge as a common sequence involving PDE5A inhibition, cGMP accumulation and smooth muscle relaxation.

The cGAS-STING pathway documented exclusively in *Epimedium brevicornu* mediates innate immune activation against tumors and neuroinflammation suppression; two seemingly opposing biological outcomes from the same pathway in the same species. This duality has not been reconciled in the reviewed studies and represents a critical knowledge gap with significant implications for therapeutic safety. Equally notable is the paraptosis mechanism reported only for epimedokoreanin B in *Epimedium koreanum*; as a non-apoptotic cell death route that bypasses classical apoptosis resistance, it has high therapeutic relevance for drug-resistant cancers, yet it rests on a single compound tested in two lung cancer lines. Finally, the bone-specific BMP-2/GLI-1/Cullin3-Nrf2 axis unique to *Epimedium pubescens* illustrates the risk of confining investigation to a single compound. While the evidence for icariin’s osteogenic properties is consistent, the absence of data on other pathways creates a falsely narrow mechanistic profile. Future studies should determine whether pathways identified in more-studied species are genuinely absent in *Epimedium grandiflorum* and *Epimedium pubescens*, or merely uninvestigated (Table 3).

## 9. Materials and Methods

### 9.1. Study Design

The manuscript was designed as a systematic review in conformity with the 2020 PRISMA guidelines. The review was intended to provide an updated, comprehensive synthesis of the phytochemistry, biological activities and therapeutic potential of five *Epimedium* species: *Epimedium grandiflorum*, *Epimedium brevicornu*, *Epimedium sagittatum*, *Epimedium koreanum*, *Epimedium pubescens*. The protocol included objectives, eligibility criteria, search strategy and synthesis approach.

### 9.2. Literature Search Strategy

A systematic literature search was conducted in three electronic databases: PubMed, Scopus and Google Scholar, to identify relevant studies published between 1991 and 2026 addressing phytochemistry, pharmacology, pharmacokinetics and toxicology of the five *Epimedium* species. The search was performed using Boolean combinations of the following keywords: “*Epimedium grandiflorum*”, “*Epimedium brevicornu*”, “*Epimedium sagittatum*”, “*Epimedium koreanum*”, “*Epimedium pubescens*”, “horny goat weed”, “prenylated flavonoids”, “icariin”, “pharmacological activity”, “phytochemistry”, “antioxidant”, “anticancer”, “pharmacokinetics” and “toxicology”.

### 9.3. Eligibility Criteria

Eligible articles included original research papers, pharmacokinetic investigations and review articles providing mechanistic context, published in English between 1991 and 2026. Studies focusing on one or more of the five targeted *Epimedium* species were prioritized. Additional studies on shared bioactive compounds such as icariin, icariside and epimedin A-C that offered relevant pharmacological or contextual support to the target species were included.

The excluded articles were duplicate studies, non-English publications and studies with no relevance to the phytochemical composition and pharmacological profile of the five *Epimedium* species. Agronomic, purely botanical or cultivation studies were also excluded unless they contained phytochemical or pharmacological data.

### 9.4. Study Selection Process

**Figure 8:**
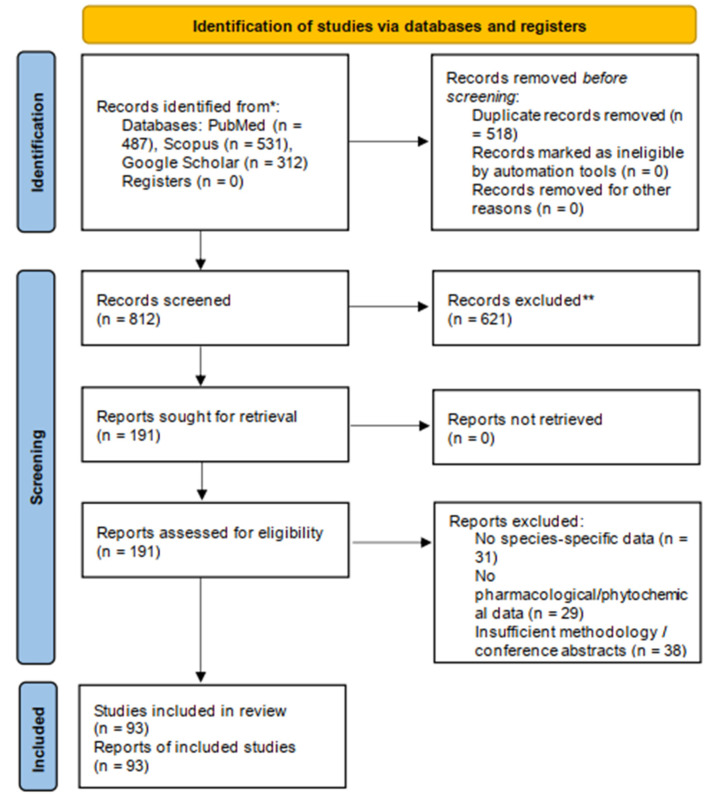
PRISMA 2020 flow diagram. * Records identified from each database (PubMed, Scopus, Google Scholar) are reported separately in the figure. ** No automation tools were used to exclude records; all screening and exclusion decisions were made manually by the authors.

The selection process was performed in line with the PRISMA 2020 framework (Figure 8).

- Stage 1—Identification: Database searches were performed by A.S.F. and I.L. Records were exported and compiled into a single reference library. Duplicates were identified and removed manually by cross-referencing titles, authors and publication years across the three databases.
- Stage 2—Screening: The titles and abstracts of the exported articles were screened for relevance. The articles that did not relate to the five *Epimedium* species or to their phytochemical and pharmacological profile were eliminated.
- Stage 3—Eligibility assessment: The full text of the remaining articles was evaluated against the predetermined inclusion and exclusion criteria. The articles that did not meet the requirements regarding content relevance, language or methodology were eliminated.
- Stage 4—Inclusion: The studies that fulfilled all the eligibility criteria were included in the present systematic review. The included studies were organized by species and the following categories: phytochemical profile, biological activities and therapeutic potential.

## 10. Conclusions

This systematic review consolidates current knowledge on the phytochemical composition, biological activities and therapeutic potential of five *Epimedium* species: *Epimedium grandiflorum* C. Morren, *Epimedium brevicornu* Maxim., *Epimedium sagittatum* (*Sieb. et Zucc.*) Maxim., *Epimedium koreanum* Nakai. and *Epimedium pubescens* Maxim. The gathered evidence confirms that prenylated flavonoids, especially icariin, icaritin and epimedin A-C represent the most important bioactive compounds. These flavonoids are responsible for the pharmacological properties attributed to the genus such as antioxidant, anti-inflammatory, immunomodulatory, neuroprotective, cardioprotective, bone-protective, androgenic and anticancer.

Although the current literature is abundant in preclinical studies, several limitations were identified across the reviewed studies. The lack of standardization, the heterogeneity of the experimental models, and the reliance on in vitro and acute in vivo models limit the ability to generalize the current findings to clinical application.

Future research should prioritize the development of standardized extraction methods and the use of chronic in vivo models that would reflect human pathology more accurately. To further validate the therapeutic potential of *Epimedium* spp. and support their integration into clinical practice, future clinical trials are needed.

## Figures and Tables

**Table 1:** Distribution of major prenylated flavonoid markers across the five reviewed *Epimedium* species.

Species	Icariin	Epimedin A	Epimedin B	Epimedin C	Icaritin	Baohuoside I	Baohuoside II	Anhydroicaritin	Desmethyl-Anhydroicaritin	Species-Specific Compounds
*E. grandiflorum*	✓	–	–	–	✓	–	–	✓	✓	Epimedigrandiosides A–B; korepimeosides A–B
*E. brevicornu*	✓	✓	✓	✓	✓	✓	✓	✓	✓	Breviflavones A–B; epimedokoreanin F; ZW1–ZW4
*E. sagittatum*	✓	✓	✓	✓	✓	✓	✓	✓	✓	Epimesatines A–S; sagittasines A–C; yinyanghuo A–E
*E. koreanum*	✓	–	✓	–	✓	–	✓	✓	✓	Korepimeosides A–C; epimedokoreanin B; koreanoside F/G
*E. pubescens*	✓	✓	✓	✓	✓	✓	✓	✓	✓	No unique markers reported to date

✓ = confirmed in published phytochemical studies; – = not reported or absent in reviewed studies.

**Table 2:** Summary of pharmacological activities, main bioactives, key mechanisms of action, and evidence levels across five *Epimedium* species.

Species	Activity	Main Bioactive	Key Mechanism(s)	Evidence Level
*E. grandiflorum*	Antioxidant	Hydroethanolic leaf extract	↑ SOD, CAT, GPx; ↓ MDA	In vitro + In vivo
	Antiproliferative	MeOH extract; prenylated flavonoids	Inhibit iNOS; G1 arrest; IC_50_ 9–45 µM	In vitro
	Androgenic/Reproductive	Hydroethanolic leaf extract	↑ Testosterone, LH, FSH; ↑ SOD; restored testicular architecture	In vitro + In vivo
	Anti-inflammatory	Epimedigrandioside A; icariside II	Inhibit NF-κB and iNOS; IC_50_ 14–26 µM	In vitro
*E. brevicornu*	Anticancer (direct)	β-Anhydroicaritin; icariside II	↑ Bax; ↓ Wnt/β-catenin; modulate TBK1/NAK	In vitro + In vivo
	Anticancer (indirect)	Icariin; epimedin C	↓ M2 macrophage polarization (PI3K/AKT); cGAS-STING → NK cytotoxicity	In vitro + In vivo
	Bone-protective	Polysaccharide; aqueous extract; nanovesicles	↑ PI3K/Akt/mTOR; ↑ Wnt/β-catenin; ↑ VEGF/VEGFR2; ↓ NLRP3/caspase-1	In vitro + In vivo
	Neuroprotective	Flavonoid extract (57% total flavonoids)	↓ PI3K/AKT + cGAS-STING (neuroinflam.); ↑ BDNF/TrkB, NRG-1/ErbB4	In vitro + In vivo
	Androgenic/Erectile	Ethanol extract; icariin	↑ eNOS; ↓ PDE5A; ↑ Testosterone; protects spermatogonial stem cells	In vitro + In vivo
*E. sagittatum*	Anti-inflammatory	Ethyl acetate extract; icariin	↓ TLR4/MD-2/NF-κB (in vitro); PI3K/AKT → Nrf2/HO-1/NQO-1 (in vivo)	In vitro + In vivo
	Neuroprotective	Icariin; icaritin	BDNF/TrkB/Akt/CREB (Alzheimer’s); PI3K/Akt + MEK/ERK (Parkinson’s)	In vitro + In vivo
	Erectile-enhancing	Icariin; baohuoside I; sagittatoside I	PDE5A1 inhibition → ↑ cGMP → ↓ Ca^2+^ → smooth muscle relaxation; ↑ eNOS	In vitro + In vivo
	Antiplatelet	Yinyanghuo A & B	Inhibit arachidonic acid-induced platelet aggregation	In vitro
*E. koreanum*	Anticancer	Aqueous extract; epimedokoreanin B & C	↓ NF-κB/MMP-9; paraptosis via ER stress; micropinocytosis (Rac1/Arf6/PIKfyve)	In vitro + In vivo
	Antioxidant	Hydroethanolic extract; polysaccharide EFPN-1	Electron delocalization via phenolic groups; Fe^2+^ chelation (EFPN-1)	In vitro
	Cardioprotective	EtOAc fraction; des-O-methyl-β-anhydroicaritin	↓ Integrin αIIbβ3; ↓ intracellular Ca^2+^; preserves HDL/apoA-I	In vitro + In vivo
	Neuroprotective	Total flavonoid fraction; magnoflorine	↑ Bcl-2/↓ Bax → dopaminergic protection; AChE inhibition → ↑ cholinergic function	In vitro + In vivo
*E. pubescens*	Anti-osteoporotic	Isolated icariin	↑ GLI-1 (osteogenesis); ↑ BMP-2 (chondrocyte diff.); ↓ Cullin3/Nrf2 → ↓ osteoclastogenesis	In vitro + In vivo

↑ upregulation/increase; ↓ downregulation/decrease; AChE, acetylcholinesterase; BDNF, brain-derived neurotrophic factor; BMP-2, bone morphogenetic protein-2; CAT, catalase; cGAS-STING, cyclic GMP-AMP synthase–stimulator of interferon genes; cGMP, cyclic guanosine monophosphate; eNOS, endothelial nitric oxide synthase; FSH, follicle-stimulating hormone; GPx, glutathione peroxidase; HDL, high-density lipoprotein; IC_50_, half-maximal inhibitory concentration; LH, luteinizing hormone; MDA, malondialdehyde; NF-κB, nuclear factor kappa-B; NK, natural killer cell; NRG-1/ErbB4, neuregulin-1/ErbB4 receptor; Nrf2/HO-1/NQO-1, nuclear factor erythroid 2-related factor 2/heme oxygenase-1/NAD(P)H quinone dehydrogenase 1; PDE5A, phosphodiesterase 5A; PI3K/AKT, phosphoinositide 3-kinase/protein kinase B; SOD, superoxide dismutase; TLR4/MD-2, Toll-like receptor 4/myeloid differentiation factor 2; TrkB, tropomyosin receptor kinase B.

**Table 3:** The distribution of key pharmacological signaling pathways documented across five *Epimedium* species.

Pathway/Mechanism	*E. gran.*	*E. brev.*	*E. sag.*	*E. kor.*	*E. pub.*
PI3K/AKT	–	✓	✓	✓	–
NF-κB inhibition	✓	–	✓	✓	–
PDE5A inhibition → ↑ cGMP	–	✓	✓	–	–
BDNF/TrkB signaling	–	✓	✓	–	–
Wnt/β-catenin	–	✓	–	–	–
Bcl-2/Bax apoptosis balance	–	✓	✓	✓	–
↑ SOD/CAT/GPx; ↓ MDA	✓	–	–	✓	–
eNOS upregulation	–	✓	✓	–	–
BMP-2/GLI-1/Cullin3-Nrf2	–	–	–	–	✓
AChE inhibition	–	–	–	✓	–
cGAS-STING activation	–	✓	–	–	–
Paraptosis/non-apoptotic death	–	–	–	✓	–

↑ upregulation/increase; ↓ downregulation/decrease; ✓ = pathway reported in reviewed studies; – = not documented. The same pathway may mediate different biological activities depending on species, compound, and experimental context. *E. gran.* = *E. grandiflorum*; *E. brev.* = *E. brevicornu*; *E. sag.* = *E. sagittatum*; *E. kor.* = *E. koreanum*; *E. pub.* = *E. pubescens*. AChE, acetylcholinesterase; Bcl-2, B-cell lymphoma-2; BDNF/TrkB, brain-derived neurotrophic factor/tropomyosin receptor kinase B; BMP-2, bone morphogenetic protein-2; cGAS-STING, cyclic GMP-AMP synthase–stimulator of interferon genes; eNOS, endothelial nitric oxide synthase; GLI-1, glioma-associated oncogene homolog 1; MDA, malondialdehyde; = NF-κB, nuclear factor kappa-B; Nrf2, nuclear factor erythroid 2-related factor 2; PDE5A, phosphodiesterase 5A; PI3K/AKT, phosphoinositide 3-kinase/protein kinase B; SOD, superoxide dismutase; Wnt/β-catenin, Wingless-related integration site/β-catenin pathway.

## Data Availability

The original contributions presented in this study are included in the article. Further inquiries can be directed to the corresponding author.

## Abbreviations

The following abbreviations are used in this manuscript:

TNF-α	Tumor necrosis factor alpha
IL-6	Interleukin-6
NF-κB	Nuclear factor kappa B
RP-18	Linear d
TLC	Thin-layer chromatography
UV	Ultraviolet
IR	Infrared
1D NMR	One-Dimensional Nuclear magnetic resonance
2D NMR	Two-Dimensional Nuclear magnetic resonance
HRESIMS	High-Resolution Electrospray Ionization Mass Spectrometry
COX	Cyclooxygenase
NO	Nitric oxide
IC_50_	Half maximal inhibitory concentration
SK-MEL	Skin melanoma cell line
KB	Human epidermoid carcinoma cell line
BT-549	Human breast carcinoma cell line
SK-OV-3	Human ovarian carcinoma cell line
LLC-PK1	Porcine kidney epithelial cell line
VERO	African green monkey kidney epithelial cell line
iNOS	Inductible nitric oxide synthase
LPS	Lipopolysaccharide
SAR	Structure-Activity Relationship
RBL-2H3	Rat Basophilic Leukemia-2H3 cell line
MAPK	Mitogen-activated protein kinase
IL-1β	Interleukin 1β
COX-2	Cyclooxygenase 2
DPPH	2.2-diphenyl-1-picrylhydrazyl
CCl_4_	Carbon tetrachloride
SOD	Superoxide dismutase
CAT	Catalase
MDA	Malondialdehyde
GPx	Glutathione peroxidase
WST-8	Water soluble tetrazolium salt-8
MCF-7	Michigan Cancer Foundation-7
LH	Luteinizing hormone
FSH	Follicle-stimulating hormone
GnRH	Gonadotropin-releasing hormone
LC-MS/MS	Liquid chromatography-tandem mass spectrometry
m/z	Mass-to-charge ratio
PDE5	Phosphodiesterase 5
mRNA	Messenger RNA
CREB	cAMP response element-binding protein
PDE3	Phosphodiesterase 3
HepG2	Human liver cancer cell line
MDA-MB-231	Human triple negative breast cancer line
Bax	Bcl-2-associated X protein
TBK1/NAK	TANK-binding kinase 1/NF-kappa-B-activating kinase
AGS	Human gastric adenocarcinoma cell line
MGC803	Human gastric cancer cell line
GES-1	Human gastric epithelial cell line
CT26-WT	Murine colorectal carcinoma cell line
HCT116	Human colorectal carcinoma cell line
AOM/DSS	Azoxymethane/Detran sodium sulfate
PI3K/AKT	Phosphoinositide 3-kinase/Protein kinase B
K562	Human chronic myelogenous leukemia cell line
NK-92	Natural killer 92 cell line
IFN-γ	Interferon gamma
NKG2D	Natural killer group 2D
Hepa1-6	Murine hepatocellular carcinoma cell line
cGAS-STING	Cyclic GMP-AMP synthase-stimulator of interferon genes
ALP	Alkaline phosphatase
mTOR	Mammalian target of rapamycin
Bcl-2	B-cell lymphoma 2
IGF-1	Insulin like growth factor 1
CTX-I	C-terminal telopeptide of type I collagen
NLRP3	NLR familypyrin domain containing 3
NPY	Neuropeptide Y
CGRP/CRLR	Calcitonin gene-related peptide/Calcitonin receptor-like receptor
VIP	Vasoactive intestinal peptide
VEGF/VEGFR2	Vascular endothelial growth factor/Vascular endothelial growth factor receptor 2
BV2	Murine microglial cell line
Aβ_42_/Aβ_1-42_	Amyloid beta 42
2VO	Two-vessel occlusion
BDNF/TrkB	Brain-derived neurotrophic factor/Tropomyosin receptor kinase B
NRG-1/ErbB4	Neuregulin 1/Erb-b2 receptor tyrosine kinase 4
CRH	Corticotropin-releasing hormone
ACTH	Adrenocorticotropic hormone
eNOS	Endothelial nitric oxide synthase
PDE5A	Phosphodiesterase 5A
H_2_O_2_	Hydrogen peroxide
SPHK1	Sphingosine kinase 1
MCF-10A	Human breast epithelial cell line
HPLC-MS/MS	High-performance liquid chromatography tandem mass spectrometry
RAW264.7	Murine macrophage cell line
TLR4/MD-2	Toll-like receptor4/Myeloid differentiation factor 2
NRK-52e	Rat kidney epithelial cell line
MCP-1	Monocyte chemoattractant protein-1
Nrf2/HO-1/NQO-1	Nuclear factor erythroid 2-related factor 2/Heme oxygenase-1/NAD(P)H quinone dehydrogenase 1
MES23.5	Murine dopaminergic cell line
MPTP	1-methyl-4-phenyl-1,2,3,6-tetrahydropyridine
MEK/ERK	Mitogen-activated protein kinase/ Extracellular signal-regulated kinase
SRC	Synapse-rich cell cluster
SMAD3	Mothers against decapentaplegic homolog 3
CEBPB	CCAAT/enhancer-binding protein beta
NR3C2	Nuclear receptor subfamily 3, group C, member 2
GPI-PLD	Glycosylphosphatidylinositol-specific phospholipase D
CCSMC	Corpus cavernosum smooth muscle cells
cGMP	Cyclic guanosine monophosphate
Ca^2+^	Calcium
AChE	Acetylcholinesterase
A172	Human glioblastoma cell line
U373MG	Human glioblastoma astrocytoma cell line
T98G	Human glioblastoma multiforme cell line
MMP-9	Matrix metallopeptidase 9
NCI-H292	Human mucoepidermoid pulmonary carcinoma cell line
A549	Human lung carcinoma cell line
Rac1	Ras-related C3 botulinum toxin substrate 1
Arf6	ADP-ribosylation factor 6
PIKfyve	Phosphoinositide kinase, FYVE-type zinc finger containing
EFPN-1	*Epimedium korenum* polysaccharide fraction 1
ATP	Adenosine triphosphate
HDL	High-density lipoprotein
apoA-I	Apolipoprotein A-I
MPP+	1-methyl-4-phenylpyridinium
DESs	Deep eutectic solvents
BMSCs	Bone mesenchymal stem cells
GLI-1	Glioma-associated oncogene Humalog 1
BMP-2	Bone morphogenetic protein 2
NFATc1	Nuclear factor of activated T-cells, cytoplasmatic 1
CtsK	Cathepsin K

## References

[1] Ekor M. (2014). The Growing Use of Herbal Medicines: Issues Relating to Adverse Reactions and Challenges in Monitoring Safety. Front. Pharmacol..

[2] Calixto J.B. (2019). The Role of Natural Products in Modern Drug Discovery. An. Acad. Bras. Cienc..

[3] Cragg G.M., Pezzuto J.M. (2016). Natural Products as a Vital Source for the Discovery of Cancer Chemotherapeutic and Chemopreventive Agents. Med. Princ. Pract..

[4] Szabó R., Rácz C.P., Dulf F.V. (2022). Bioavailability Improvement Strategies for Icariin and Its Derivates: A Review. Int. J. Mol. Sci..

[5] Sayed Salman S., Priyanka B., Sowmya Latha Sri G., Boddeda B. (2025). Emerging Trends in Advanced Herbal Pharmaceuticals: From Bench to Bedside. GSC Biol. Pharm. Sci..

[6] Newman D.J., Cragg G.M. (2020). Natural Products as Sources of New Drugs over the Nearly Four Decades from 01/1981 to 09/2019. J. Nat. Prod..

[7] Yang Y.H., Mao J.W., Tan X.L. (2020). Research Progress on the Source, Production, and Anti-Cancer Mechanisms of Paclitaxel. Chin. J. Nat. Med..

[8] Babashpour-Asl M., Kaboudi P.S., Barez S.R. (2023). Therapeutic and Medicinal Effects of Snowdrop (*Galanthus* spp.) in Alzheimer’s Disease: A Review. J. Educ. Health Promot..

[9] de Swiet M. (2023). Digitalis Purpurea the Source of Digitoxin: Digitalis Lanata the Source of Digoxin. Modern Medicines from Plants.

[10] Foster G. (2023). Papaver Somniferum: The Source of Morphine, Codeine, Noscapine, Protopine, Papaverine and Verapamil. Modern Medicines from Plants.

[11] Ho J.F.V., Yaakup H., Low G.S.H., Wong S.L., Tho L.M., Tan S.B. (2020). Morphine Use for Cancer Pain: A Strong Analgesic Used Only at the End of Life? A Qualitative Study on Attitudes and Perceptions of Morphine in Patients with Advanced Cancer and Their Caregivers. Palliat. Med..

[12] Lee B.J., Weyers M., Haynes R.K., van der Kooy F. (2023). Discovery of Artemisinin in Artemisia Annua, Its Current Production, and Relevance to Sub-Saharan Africa. S. Afr. J. Bot..

[13] de Swiet M. (2023). Cinchona: The Source of Quinine and Quinidine. Modern Medicines from Plants.

[14] Xie S.S., Yu X., Tie Q.M., Zhang J.K., Zhang B.B., Zeng M.N., Zheng X.K., Feng W.S. (2025). Six Pairs of Enantiomeric Prenylated Flavonoids with Cytotoxic Activities from Epimedium Sagittatum Maxim. Nat. Prod. Bioprospecting.

[15] Cui J., Lin L., Hao F., Shi Z., Gao Y., Yang T., Yang C., Wu X., Gao R., Ru Y. (2024). Comprehensive Review of the Traditional Uses and the Potential Benefits of Epimedium Folium. Front. Pharmacol..

[16] Wang Y., Han Y., Zhu H., Xia P. (2024). A Systematic Review of the Botany, Traditional Uses, Phytochemistry and Pharmacology of Epimedium. Phytochem. Rev..

[17] Ma H., He X., Yang Y., Li M., Hao D., Jia Z. (2011). The Genus Epimedium: An Ethnopharmacological and Phytochemical Review. J. Ethnopharmacol..

[18] Luo Z., Dong J., Wu J. (2022). Impact of Icariin and Its Derivatives on Inflammatory Diseases and Relevant Signaling Pathways. Int. Immunopharmacol..

[19] Reyes-Hernández O.D., Figueroa-González G., Quintas-Granados L.I., Hernández-Parra H., Peña-Corona S.I., Cortés H., Kipchakbayeva A., Mukazhanova Z., Habtemariam S., Leyva-Gómez G. (2024). New Insights into the Anticancer Therapeutic Potential of Icaritin and Its Synthetic Derivatives. Drug Dev. Res..

[20] Chen X.L., Li S.X., Ge T., Zhang D.D., Wang H.F., Wang W., Li Y.Z., Song X.M. (2024). Epimedium Linn: A Comprehensive Review of Phytochemistry, Pharmacology, Clinical Applications and Quality Control. Chem. Biodivers..

[21] Lee E.L., Barnes J. (2025). Horny Goat Weed/Epimedium. J. Prim. Health Care.

[22] He C., Wang Z., Shi J. (2020). Pharmacological Effects of Icariin. Adv. Pharmacol..

[23] Lv H.W., Wang Q.L., Luo M., Zhu M.D., Liang H.M., Li W.J., Cai H., Zhou Z.B., Wang H., Tong S.Q. (2023). Phytochemistry and Pharmacology of Natural Prenylated Flavonoids. Arch. Pharmacal Res..

[24] Basabe P., De Román M., Marcos I.S., Diez D., Blanco A., Bodero O., Mollinedo F., Sierra B.G., Urones J.G. (2010). Prenylflavonoids and Prenyl/Alkyl-Phloroacetophenones: Synthesis and Antitumour Biological Evaluation. Eur. J. Med. Chem..

[25] Gani I., Jameel S., Bhat S.A., Amin H., Bhat K.A. (2023). Prenylated Flavonoids of Genus Epimedium: Phytochemistry, Estimation and Synthesis. ChemistrySelect.

[26] Shi S., Li J., Zhao X., Liu Q., Song S.J. (2021). A Comprehensive Review: Biological Activity, Modification and Synthetic Methodologies of Prenylated Flavonoids. Phytochemistry.

[27] Zulfiqar F., Khan S.I., Ross S.A., Ali Z., Khan I.A. (2017). Prenylated Flavonol Glycosides from Epimedium Grandiflorum: Cytotoxicity and Evaluation against Inflammation and Metabolic Disorder. Phytochem. Lett..

[28] Samra R.M., Othman A., Elsbaey M., Amen Y., Shimizu K. (2024). Comprehensive Review on Megastigmane Glycosides: Sources, Bioactivities, and 13C NMR Spectroscopic Data. Phytochem. Lett..

[29] Shu P., Wei X., Xue Y., Li W., Zhang J., Xiang M., Zhang M., Luo Z., Li Y., Yao G. (2013). Wilsonols A-L, Megastigmane Sesquiterpenoids from the Leaves of Cinnamomum Wilsonii. J. Nat. Prod..

[30] El-Sayed H.M., Rasheed D.M., Mahrous E.A., Abdel-Sattar E. (2025). C13-Norisoprenoid Megastigmanes: Biosynthesis, Classification, Natural Sources, Biological Activities, and Structure-Activity Relationship–A Comprehensive Review. Fitoterapia.

[31] Imahori D., Μatsumoto T., Saito Y., Ohta T., Yoshida T., Nakayama Y., Watanabe T. (2021). Cell Death-Inducing Activities via P-Glycoprotein Inhibition of the Constituents Isolated from Fruits of Nandina Domestica. Fitoterapia.

[32] Goda Y., Hoshino K., Akiyama H., Ishikawa T., Abe Y., Nakamura T., Otsuka H., Takeda Y., Tanimura A., Toyoda M. (1999). Constituents in Watercress: Inhibitors of Histamine Release from RBL-2H3 Cells Induced by Antigen Stimulation. Biol. Pharm. Bull..

[33] Alam M.B., Kwon Y.G., Simu S.Y., Shahriyar S.A., Lee S.H. (2020). Attenuation of Inflammatory Symptoms by Icariside B2 in Carrageenan and LPS-Induced Inflammation Models via Regulation of MAPK/NF-ΚB Signaling Cascades. Biomolecules.

[34] Asif N., Amjad S., Hussein K., Salman M., Qureshi F., Bukhari I.N., Khalid R., Zahid F. (2016). Physicochemical Characterization and Antioxidant Activity of Extract of Epimedium Grandiflorum. J. Pharm. Chem..

[35] Munir N., Mahmood Z., Yameen M., Mustafa G. (2020). Therapeutic Response of *Epimedium gandiflorum*’s Different Doses to Restore the Antioxidant Potential and Reproductive Hormones in Male Albino Rats. Dose-Response.

[36] Ilkhani E., Asghari A., Mortazavi P., Hassanpour S. (2025). Original Article Evaluating the Antioxidant Potential of Epimedium Grandiflorum in a Rat Model of Cryptorchidism: Reducing Malondialdehyde and Enhancing Antioxidant Enzymes. Iran. J. Vet. Med..

[37] Telang N.T., Li G., Katdare M., Sepkovic D.W., Bradlow H.L., Wong G.Y.C. (2017). The Nutritional Herb Epimedium Grandiflorum Inhibits the Growth in a Model for the Luminal a Molecular Subtype of Breast Cancer. Oncol. Lett..

[38] Corazza O., Martinotti G., Santacroce R., Chillemi E., Di Giannantonio M., Schifano F., Cellek S. (2014). Sexual Enhancement Products for Sale Online: Raising Awareness of the Psychoactive Effects of Yohimbine, Maca, Horny Goat Weed, and *Ginkgo biloba*. BioMed Res. Int..

[39] Nasef A.Z., El-Sheikh N.A. (2023). Potential Effects of Horny Goat Weed (Epimedium Grandiflorum) on The Level of Fertility in Male Rats Infected with Cadmium Chloride: Biochemical and Histopathological Study. Alex. Sci. Exch. J..

[40] Musa R.F., Obeid A.K. (2025). Protective Role of Epimedium Grandiflorum Extract on Epididymal Tissue and Sperm Parameters in Male Rats Treated with Cisplatin. J. Kerbala Agric. Sci..

[41] Evaluation of the Protective Role of Epimedium Grandiflorum Leaf Nano-Extract in Testicular Histological Changes in Male Albino Rats Treated with Amlodipine–Arab Democratic Center. https://democraticac.de/?p=109369.

[42] Yap S.P., Shen P., Butler M.S., Gong Y., Loy C.J., Yong E.L. (2005). New Estrogenic Prenylflavone from Epimedium Brevicornum Inhibits the Growth of Breast Cancer Cells. Planta Med..

[43] Shen P., Guo B.L., Gong Y., Hong D.Y.Q., Hong Y., Yong E.L. (2007). Taxonomic, Genetic, Chemical and Estrogenic Characteristics of Epimedium Species. Phytochemistry.

[44] Hong X., Wang X., Yong E.L., Gong Y. (2009). Determination of Breviflavone A and B in Epimedium Herbs with Liquid Chromatography–Tandem Mass Spectrometry. J. Pharm. Biomed. Anal..

[45] Chen Y.C., Zheng Q., Lu H., Zhuang Y.X., Gong T.T., Ma L.F., Zhan Z.J. (2023). Neuroprotective Flavonoids from Epimedium Brevicornu by Inhibition of Ferroptosis. Phytochem. Lett..

[46] Li F., Du B.W., Lu D.F., Wu W.X., Wongkrajang K., Wang L., Pu W.C., Liu C.L., Liu H.W., Wang M.K. (2017). Flavonoid Glycosides Isolated from Epimedium Brevicornum and Their Estrogen Biosynthesis-Promoting Effects. Sci. Rep..

[47] Pang X., Yin S.S., Yu H.Y., Zhang Y., Wang T., Hu L.M., Han L.F. (2018). Prenylated Flavonoids and Dihydrophenanthrenes from the Leaves of Epimedium Brevicornu and Their Cytotoxicity against HepG2 Cells. Nat. Prod. Res..

[48] Wang X., Cui B., Xu L., Pei X. (2024). Exploring the Molecular Mechanism of Epimedium Brevicornu Maxim. in Treating Breast Cancer via Network Pharmacology and in Vitro Experiments. J. Tradit. Chin. Med. Sci..

[49] Dou R., Zhu X., Liu X., Bao J., Jin R., Mao G., Yu H., Liu Y. (2025). Icariside II Inhibits Gastric Cancer Progression by Suppressing the Wnt/β-Catenin Signaling Pathway. Cytotechnology.

[50] Chen Y., Qi Y., Jiang Y., Li Y., Yang S., Wang L., Li M., Chai K., Wang Y. (2025). Icariin Modulates the Tumor Microenvironment in Colorectal Cancer by Targeting M2 Macrophage Polarization via PI3K/AKT Pathway. Bioorg. Med. Chem..

[51] Liu L., Zhan X., Wang X., Wen J., He C., Chen X., Guo Y., Wang X., Li L., Cheng H. (2025). Epimedium Brevicornu Maxim. Extract Activates Natural Killer Cells against Hepatocellular Carcinoma via the CGAS-STING Pathway. Front. Pharmacol..

[52] Zheng H., He B., Wu T., Cai J., Wei J. (2020). Extraction, Purification and Anti-Osteoporotic Activity of a Polysaccharide from Epimedium Brevicornum Maxim. in Vitro. Int. J. Biol. Macromol..

[53] Ma F., Zhang W., Zhou G., Qi Y., Mao H.-R., Chen J., Lu Z., Wu W., Zou X., Deng D. (2024). Epimedii Folium Decoction Ameliorates Osteoporosis in Mice through NLRP3/Caspase-1/IL-1β Signalling Pathway and Gut-Bone Axis. Int. Immunopharmacol..

[54] Liu H., Xiong Y., Wang H., Yang L., Wang C., Liu X., Wu Z., Li X., Ou L., Zhang R. (2018). Effects of Water Extract from Epimedium on Neuropeptide Signaling in an Ovariectomized Osteoporosis Rat Model. J. Ethnopharmacol..

[55] Cai X., Qiu T., Shen J., Xu Y., Wu Z., Lin J., Yang H., Zhao Q., Zhao K. (2025). Epimedium Brevicornu Maxim.-Derived Extracellular Vesicle-like Particles Stimulate VEGF-Mediated Angiogenesis to Alleviate Postmenopausal Osteoporosis. Phytomedicine.

[56] Li S., Xiao D., Yusufu R., Huang F., Feng Y., Abudureyimu A., Zhao J., Jiang H., Wei F. (2026). Epimedium Brevicornu Flavonoids Alleviate Neuroinflammation and Alzheimer’s Disease Pathology via Immune-Related Pathways. Phytomedicine.

[57] Niu H.-M., Wang M.-Y., Ma D.-L., Chen X.-P., Zhang L., Li Y.-L., Zhang L., Li L. (2020). Epimedium Flavonoids Improve Cognitive Impairment and White Matter Lesions Induced by Chronic Cerebral Hypoperfusion through Inhibiting the Lingo-1/Fyn/ROCK Pathway and Activating the BDNF/NRG1/PI3K Pathway in Rats. Brain Res..

[58] Wang K., Li J., Zheng X., Xu J., Wang Z., Li S., Yang Q., Wu Y., Yang D.H., Yao S. (2023). The Pharmacological Effects and Safety of the Raw and Prepared Folium of Epimedium Brevicornu Maxim. on Improving Kidney-Yang Deficiency Syndrome and Sexual Dysfunction. Front. Pharmacol..

[59] Liao T.L., He C.M., Xiao D., Zhang Z.R., He Z., Yang X.P. (2025). Icariin Targets PDE5A to Regulate Viability, DNA Synthesis and DNA Damage of Spermatogonial Stem Cells and Improves Reproductive Capacity. Asian J. Androl..

[60] Matsushita H., Miyase T., Ueno A. (1991). Lignan and Terpene Glycosides from Epimedium Sagittatum. Phytochemistry.

[61] Wang G.J., Tsai T.H., Lin L.C. (2007). Prenylflavonol, Acylated Flavonol Glycosides and Related Compounds from Epimedium Sagittatum. Phytochemistry.

[62] Miyase T., Ueno A. (1991). Ionone and Bibenzyl Glycosides from Epimedium Grandiflorum Var. Thunbergianum. Phytochemistry.

[63] Xie S., Zeng M., Zhang J., Liu J., Wei J., Wang R., Li M., Hao Z., Ji B., Zheng X. (2022). Epimesatines A–I, Nine Undescribed Prenylated Flavonoids with SPHK1 Inhibitory Activities from Epimedium Sagittatum Maxim. Phytochemistry.

[64] Xie S.S., Yu X., Zhang J.K., Hao Z.Y., Zheng X.K., Feng W.S. (2024). Epimesatines P–S: Four Undescribed Flavonoids from Epimedium Sagittatum Maxim. and Their Cytotoxicity Activities. Molecules.

[65] Chen C.C., Huang Y.L., Sun C.M., Shen C.C., Ko F.N., Teng C.M. (1996). New Prenylflavones from the Leaves of Epimedium Sagittatum. J. Nat. Prod..

[66] Lee P.H., Liu C.M., Huang S.T., Li W.J., Li H.T., Chen C.Y. (2026). A New Tetraazacyclododecane of Epimedium Sagittatum. Chem. Nat. Compd..

[67] Chen C.Y., Liu C.M., Yeh H.C., Li W.J., Li H.T., Cheng M.J. (2022). A New β-Ionone from Epimedium Sagittatum. Chem. Nat. Compd..

[68] Yan N., Wen D.S., Zhao Y.R., Xu S.J. (2018). Epimedium Sagittatum Inhibits TLR4/MD-2 Mediated NF-ΚB Signaling Pathway with Anti-Inflammatory Activity. BMC Complement. Altern. Med..

[69] Wang R., Zeng M., Zhang B., Zhang Q., Xie S., Hu Y., Fan R., Wang M., Yu X., Zhang Y. (2023). Epimedium Sagittatum Maxim Ameliorates Adriamycin-Induced Nephropathy by Restraining Inflammation and Apoptosis via the PI3K/AKT Signaling Pathway. Immun. Inflamm. Dis..

[70] Sheng C., Xu P., Zhou K., Deng D., Zhang C., Wang Z. (2017). Icariin Attenuates Synaptic and Cognitive Deficits in an Aβ1–42-Induced Rat Model of Alzheimer’s Disease. BioMed Res. Int..

[71] Wu H., Zhang Z.H., Zhou P., Sui X., Liu X., Sun Y., Zhao X., Pu X.P. (2024). A Single-Cell Atlas of the Substantia Nigra Reveals Therapeutic Effects of Icaritin in a Rat Model of Parkinson’s Disease. Antioxidants.

[72] Li J., Wu Y., Yu X., Zheng X., Xian J., Li S., Shi W., Tang Y., Chen Z.S., Liu G. (2022). Isolation, Bioassay and 3D-QSAR Analysis of 8-Isopentenyl Flavonoids from Epimedium Sagittatum Maxim. as PDE5A Inhibitors. Chin. Med..

[73] Fang Y., Wu S., Xu J., Chen J., Shi W., Wang H., Li S., Xie H., Tao H., Pan L. (2026). Mechanistic Study of PDE5A Inhibitors from the Prepared Folium of Epimedium Sagittatum Maxim. J. Struct. Biol..

[74] Li J., He Y., Zheng X., Li S., Wu Y., Shi W., Pan K., Sun J., Wang Z., Xu J. (2023). Flavonoids and Prenylhydroquinones from the Prepared Folium of Epimedium Sagittatum Maxim. and Their Inhibition against Phosphodiesterase5A. Fitoterapia.

[75] Zheng X., Li S., Wang K., Wang Z., Li J., Yang Q., Wu Y., Chen Q., Dou Y., Yao S. (2024). Comparing the Pharmacological Effects of the Prepared Folium of Epimedium Brevicornu Maxim. and Epimedium Sagittatum Maxim. on Kidney-Yang Deficiency Syndrome and Liver Injury Complications. Fitoterapia.

[76] Li J.Y., Li H.M., Liu D., Chen X.Q., Chen C.H., Li R.T. (2016). Three New Acylated Prenylflavonol Glycosides from Epimedium Koreanum. Phytochem. Lett..

[77] Zhang H., Wu X., Wang J., Wang M., Wang X., Shen T., Wang S., Ren D. (2020). Flavonoids from the Leaves of Epimedium Koreanum Nakai and Their Potential Cytotoxic Activities. Nat. Prod. Res..

[78] Zhang X., Oh M., Kim S., Kim J., Kim H., Kim S., Houghton P.J., Whang W. (2013). Epimediphine, a Novel Alkaloid from Epimedium Koreanum Inhibits Acetylcholinesterase. Nat. Prod. Res..

[79] Lee W., Nam J.H., Cho H.J., Lee J.Y., Cho W.K., Kim Y., We Y.M., Ma J.Y., Hoe H.S. (2017). Epimedium Koreanum Nakai Inhibits PMA-Induced Cancer Cell Migration and Invasion by Modulating NF-ΚB/MMP-9 Signaling in Monomorphic Malignant Human Glioma Cells. Oncol. Rep..

[80] Liu X., Wang S., Zheng H., Liu Q., Shen T., Wang X., Ren D. (2021). Epimedokoreanin C, a Prenylated Flavonoid Isolated from Epimedium Koreanum, Induces Non-Apoptotic Cell Death with the Characteristics of Methuosis in Lung Cancer Cells. Am. J. Cancer Res..

[81] Zheng H., Liu Q., Wang S., Liu X., Ma M., Shen T., Wang X., Ren D. (2022). Epimedokoreanin B Inhibits the Growth of Lung Cancer Cells through Endoplasmic Reticulum Stress-Mediated Paraptosis Accompanied by Autophagosome Accumulation. Chem. Biol. Interact..

[82] Zhao Y., Chen S., Wang Y., Lv C., Wang J., Lu J. (2018). Effect of Drying Processes on Prenylflavonoid Content and Antioxidant Activity of Epimedium Koreanum Nakai. J. Food Drug Anal..

[83] Li B., Zhang N., Wang D.X., Jiao L., Tan Y., Wang J., Li H., Wu W., Jiang D.C. (2018). Structural Analysis and Antioxidant Activities of Neutral Polysaccharide Isolated from Epimedium Koreanum Nakai. Carbohydr. Polym..

[84] Irfan M., Kwon T.H., Lee D.H., Hong S.B., Oh J.W., Kim S.D., Rhee M.H. (2021). Antiplatelet and Antithrombotic Effects of Epimedium Koreanum Nakai. Evid. Based Complement. Altern. Med..

[85] Kim J.Y., Shim S.H. (2019). Epimedium Koreanum Extract and Its Flavonoids Reduced Atherosclerotic Risk via Suppressing Modification of Human HDL. Nutrients.

[86] Wu L., Du Z.R., Xu A.L., Yan Z., Xiao H.H., Wong M.S., Yao X.S., Chen W.F. (2017). Neuroprotective Effects of Total Flavonoid Fraction of the Epimedium Koreanum Nakai Extract on Dopaminergic Neurons: In Vivo and in Vitro. Biomed. Pharmacother..

[87] Jeong J., Whang W., Oh M. (2026). Improvement of Cognitive Impairment and Isolation of the Active Compound in the Water Fraction of *Epimedium Koreanum* MeOH Extract. Yakhak Hoeji.

[88] Zheng H., Zheng L., Yu X. (2025). Non-Targeted Metabolomics Study of *Epimedium pubescens* in Response to Light Stress. J. Plant Growth Regul..

[89] Wang Y., Peng B., Zhao J., Wang M., Zhao L. (2020). Efficient Extraction and Determination of Prenylflavonol Glycosides in *Epimedium pubescens* Maxim. Using Deep Eutectic Solvents. Phytochem. Anal..

[90] Xia S.L., Ma Z.Y., Wang B., Gao F., Guo S.Y., Chen X.H. (2023). Icariin Promotes the Proliferation and Osteogenic Differentiation of Bone-Derived Mesenchymal Stem Cells in Patients with Osteoporosis and T2DM by Upregulating GLI-1. J. Orthop. Surg. Res..

[91] Iqbal M., Zhang H., Mehmood K., Li A., Jiang X., Wang Y., Zhang J., Iqbal M.K., Rehman M.U., Yao W. (2018). Icariin: A Potential Compound for the Recovery of Tibial Dyschondroplasia Affected Chicken Via Up-Regulating BMP-2 Expression. Biol. Proced. Online.

[92] Si Y., Li Y., Gu K., Yin H., Ma Y. (2024). Icariin Ameliorates Osteoporosis in Ovariectomized Rats by Targeting Cullin 3/Nrf2/OH Pathway for Osteoclast Inhibition. Biomed. Pharmacother..

